# A conserved tryptophan in the acylated segment of RTX toxins controls their β_2_ integrin–independent cell penetration

**DOI:** 10.1016/j.jbc.2023.104978

**Published:** 2023-06-28

**Authors:** Adriana Osickova, Sarka Knoblochova, Ladislav Bumba, Petr Man, Zuzana Kalaninova, Anna Lepesheva, David Jurnecka, Monika Cizkova, Lada Biedermannova, Jory A. Goldsmith, Jennifer A. Maynard, Jason S. McLellan, Radim Osicka, Peter Sebo, Jiri Masin

**Affiliations:** 1 Institute of Microbiology of the Czech Academy of Sciences, Prague, Czech Republic; 2 Faculty of Sciences, Charles University, Prague, Czech Republic; 3 Institute of Biotechnology of the Czech Academy of Sciences, BIOCEV, Vestec, Czech Republic; 4 Department of Molecular Biosciences, The University of Texas at Austin, Austin, USA; 5 Department of Chemical Engineering, The University of Texas at Austin, Austin, USA

**Keywords:** RTX toxin, adenylate cyclase toxin, α-hemolysin, β_2_ integrins, acylated segment, tryptophan residue, thermal stability, hydrogen/deuterium exchange, cytotoxicity

## Abstract

The acylated Repeats in ToXins (RTX) leukotoxins, the adenylate cyclase toxin (CyaA) or α-hemolysin (HlyA), bind β_2_ integrins of leukocytes but also penetrate cells lacking these receptors. We show that the indoles of conserved tryptophans in the acylated segments, W876 of CyaA and W579 of HlyA, are crucial for β_2_ integrin–independent membrane penetration. Substitutions of W876 by aliphatic or aromatic residues did not affect acylation, folding, or the activities of CyaA W876L/F/Y variants on cells expressing high amounts of the β_2_ integrin CR3. However, toxin activity of CyaA W876L/F/Y on cells lacking CR3 was strongly impaired. Similarly, a W579L substitution selectively reduced HlyA W579L cytotoxicity towards cells lacking β_2_ integrins. Intriguingly, the W876L/F/Y substitutions increased the thermal stability (T_*m*_) of CyaA by 4 to 8 °C but locally enhanced the accessibility to deuteration of the hydrophobic segment and of the interface of the two acylated loops. W876Q substitution (showing no increase in T_*m*_), or combination of W876F with a cavity-filling V822M substitution (this combination decreasing the T_*m*_ closer to that of CyaA), yielded a milder defect of toxin activity on erythrocytes lacking CR3. Furthermore, the activity of CyaA on erythrocytes was also selectively impaired when the interaction of the pyrrolidine of P848 with the indole of W876 was ablated. Hence, the bulky indoles of residues W876 of CyaA, or W579 of HlyA, rule the local positioning of the acylated loops and enable a membrane-penetrating conformation in the absence of RTX toxin docking onto the cell membrane by β_2_ integrins.

Repeats in ToXins (RTX) of Gram-negative pathogens form a diverse family of cytotoxic proteins that primarily target leukocytes to subvert host immune defense (1, 2, 3). The shared feature of RTX toxins is their unique mode of export from the bacterial cytosol across the bacterial cell envelope through the type I secretion system, which recognizes an unprocessed C-terminal secretion signal of the calcium-binding RTX nonapeptide repeat domain. Folding and biological activity of the RTX toxins requires millimolar concentration of free calcium ions and an acyltransferase-mediated covalent posttranslational fatty acylation of two internal lysine residues within the acylated segment (2, 4, 5, 6, 7). The RTX adenylate cyclase toxin hemolysin (CyaA) of the pertussis agent *Bordetella pertussis* primarily targets myeloid phagocytes. The toxin inserts into the plasma membrane of cells and delivers its N-terminal adenylyl cyclase (AC) enzyme domain (∼400 residues) directly from lipid rafts into the cell cytosol (8, 9). The RTX hemolysin (Hly) portion of CyaA (∼1300 C-proximal residues) can also function independently, and its N-terminal hydrophobic pore-forming segment (PF, residues ∼500 to ∼700) forms small oligomeric cation-selective membrane pores (10, 11). The Hly portion of CyaA further comprises a large RTX domain (RTX) consisting of five blocks of glycine- and aspartate-rich nonapeptide repeats that form calcium-binding β-rolls separated by conserved linkers (12, 13). The N-terminal β-roll extension of the RTX domain (acylated segment, residues 750–1000) harbors two conserved acylation sites on lysine residues K860 and K983, which are covalently palmitoylated on their ε-amino groups by the co-expressed toxin-activating acyltransferase CyaC (14, 15).

Another prototypical pore-forming RTX cytolysin is the α-hemolysin (HlyA) of *Escherichia coli* (2). Its N-terminal ∼200 amino acid residues, preceding the pore-forming hydrophobic domain, are predicted to contain multiple amphipathic α-helices, whereas the C-terminal calcium-binding RTX domain of HlyA is then predicted to consist of an extended β-roll block of nonapeptide repeats. The acylated segment of HlyA (residues ∼450 to ∼720) includes two acylated lysines, K564 and K690, modified by HlyC (16, 17).

Several RTX toxins, including CyaA and HlyA, utilize as cellular receptors the highly glycosylated β_2_ integrins of leukocytes, in which a specific α_L_ (CD11a), α_M_ (CD11b), α_X_ (CD11c), or α_D_ (CD11d) subunit forms a heterodimeric complex with a shared CD18 β_2_ subunit (18). Several members of the RTX toxin family, such as HlyA or *Aggregatibacter actinomycetemcomitans* LtxA, *Mannheimia haemolytica* LktA, and *Actinobacillus pleuropneumoniae* ApxIIIA, exclusively engage the β_2_ subunit, making the toxins bind to all β_2_-containing heterodimers (19). In contrast, the CyaA toxin selectively binds the α_M_ (CD11b) subunit of the α_M_β_2_ integrin, known as the complement receptor 3 (CR3, Mac-1, or CD11b/CD18) (20, 21). Nevertheless, due to promiscuous low-affinity binding to glycan moieties of cellular gangliosides and glycoproteins, the CyaA and HlyA toxins can also bind and penetrate with reduced potency all kinds of cells lacking β_2_ integrins, such as erythrocytes or epithelial cells (22, 23, 24).

Recently, the structure of the CyaA RTX domain–CR3 complex was solved by cryo-EM analysis (12, 13), complementing the previously reported structures of the AC, ‘AC-to-Hly linker,’ and RTX blocks IV/V (4, 25, 26, 27). Nonetheless, the structure-function relationships underlying the AC-translocating activity of the membrane-penetrating domain of CyaA (residues 400–700) remain poorly understood. Despite inferences from the functional characterization of mutant toxin variants, a mechanistic understanding of CyaA intoxication remains to be defined (9, 28, 29, 30, 31, 32, 33, 34, 35, 36). To partially address this gap, we report that the highly conserved tryptophan residues of CyaA (W876) and HlyA (W579) fine-tune the structural configuration of acylated segments of RTX leukotoxins to enable their integrin receptor-independent (nonassisted) membrane penetration.

## Results

### The indole side chain of W876 is selectively required for cytotoxic activity of CyaA on CR3-deficient cells

**Figure 1:**
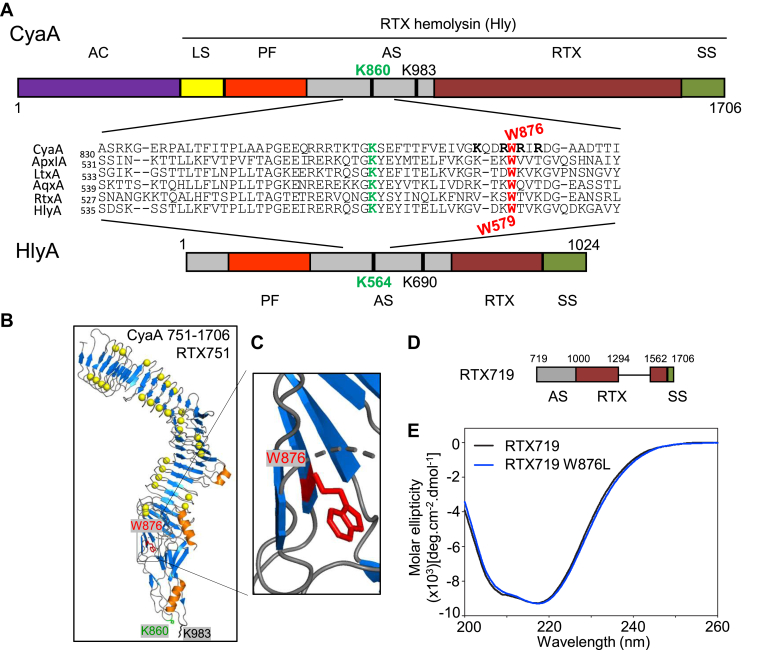
**Schematic representation of the domain architecture of CyaA and HlyA.***A*, CyaA consists of an adenylyl cyclase AC enzyme domain (AC) and an RTX hemolysin moiety (Hly). The latter contains an ‘AC-to-Hly’ linker segment (LS), a pore-forming domain (PF), an acylated segment (AS) containing palmitoylated K860 and K983 residues, a calcium-loaded and CR3-binding RTX domain (RTX), and a C-terminal secretion signal (SS). HlyA consists of the hydrophobic pore-forming domain at the N-terminus and the RTX repeats domain at the C-terminus. The acylated segment containing K564 and K690 is in the central part of HlyA between residues ∼450 and 720. The zoomed-out section shows the ClustalW sequence alignments of portions of the acylated segments harboring the first acylated lysine residues (in *bold green* color) of RTX toxins. CyaA, *Bordetella pertussis* (UniProtKB P0DKX7); ApxIA, *Actinobacillus pleuropneumoniae* (UniProtKB P55128); LtxA, *Aggregatibacter actinomycetemcomitans* (UniProtKB P16462); AqxA, *Actinobacillus equuli* (UniProtKB Q8KWZ9); RtxA, *Kingella kingae* (UniProtKB A0A1X7QMH9); HlyA, *Escherichia coli* (UniProtKB A0A4Z0T8K2). Conserved tryptophans are highlighted in *bold red*; acylated lysines are highlighted in *bold green*. *B*, PyMol view of RTX751 (13), (CyaA 751–1706, PDB 7USL). The tryptophan 876 residue is highlighted in *red* color, and calcium ions are shown as *yellow spheres*. *C*, close-up view of the relevant part of the acylated segment of CyaA. *D*, schematic representation of the CyaA-derived RTX719 construct. *E*, far-UV CD spectra of the RTX719 variants (200 μg/ml) refolded from 8 M urea in 5 mM Tris–HCl (pH 8.0), 20 mM NaCl containing 2 mM CaCl_2_. AC, adenylyl cyclase; CR3, complement receptor 3; Hly, hemolysin domain; HlyA, α-hemolysin; RTX, Repeats in ToXins blocks.

**Figure 2:**
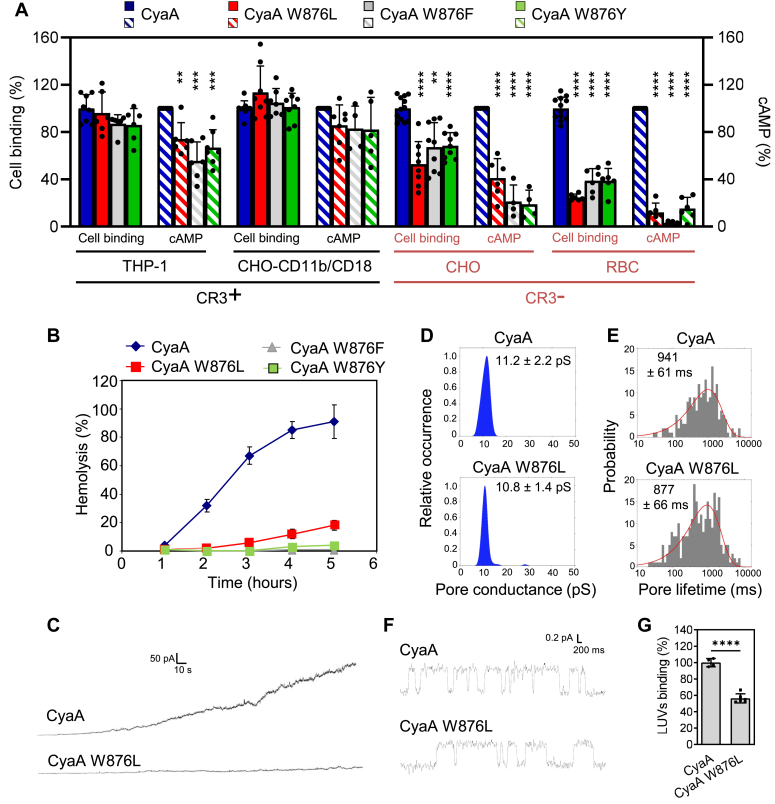
**The indole side chain of W876 is required for toxin activity of CyaA on cells lacking the CR3 receptor.***A*, cell binding (*filled* bars) and cAMP intoxication (*hatched* bars) of CyaA toxin variants was determined on human CR3-expressing THP-1 monocytes, on CR3-expressing CHO-CD11b/CD18 transfectants and on mock-transfected CHO cells (CHO) or sheep erythrocytes (RBC) that do not express the CR3 receptor. Toxin binding to THP-1, CHO-CD11b/CD18, and mock CHO cells was determined as the amount of total cell-associated AC enzyme activity after incubation of 10^6^ cells with 1 μg/ml of the protein for 30 min at 4 °C. For assays on erythrocytes, 5 × 10^8^ RBC were incubated with 1 μg/ml of the indicated toxin for 30 min at 37 °C prior to determination of cell-associated AC enzyme activity. AC translocation was assessed by determining the intracellular cAMP concentration as described in Experimental procedures. Activities are expressed as a percentage of CyaA activity and represent the means ± SD of at least four independent determinations performed in duplicate with two different toxin preparations. *B*, hemolytic activity was measured as the amount of hemoglobin released from erythrocytes (5 × 10^8^/ml) after incubation with CyaA (5 μg/ml) using a photometric assay (A_541nm_). *C*, overall membrane activity of 1.5 nM CyaA and CyaA W876L on planar lipid bilayer membranes made of L-α-phosphatidylcholine and bathing in 50 mM KCl, 10 mM Tris–HCl (pH 7.4), 2 mM CaCl_2_, with 50 mV applied voltage at 25 °C. The recordings were filtered at 10 Hz. The increase of membrane current (pA) in time (s) was measured. *D*, kernel density estimation of single-pore conductances calculated from single-pore conductance recordings (>500 events) acquired on different membranes exposed to 500 pM CyaA variants under the same conditions as in (*C*). *E*, for lifetime determination, >250 individual pore openings were recorded on different membranes exposed to 500 pM CyaA under the same conditions as in (*C*). Numbers in panels *D* and *E* represent the most frequent values ± S.D. *F*, recording of single pore conductance occurring in asolectin membranes exposed to 500 pM CyaA variants under otherwise identical conditions as in (*C*). *G*, binding of CyaA to 100 nm LUVs made of L-α-phosphatidylcholine was determined as the amount of total LUV-associated AC enzyme activity following LUV incubation with 1 μg/ml of CyaA for 15 min at 37 °C. Insertion of CyaA into lipid membrane of LUVs was determined after repeated washing and stripping of noninserted toxin with 0.1 M sodium carbonate pH 10.5, as described under Experimental procedures. Binding of CyaA W876L to liposomes was expressed as a percentage of intact CyaA activity and represents mean values ± SD of three independent determinations performed in duplicate. Statistical significance was determined using the one-way ANOVA test. ∗∗∗∗*p* < 0.0001, ∗∗∗*p* < 0.001, ∗∗*p* < 0.01. CR3^+^; CR3-expressing cells, CR3^−^; cells not expressing CR3. AC, adenylyl cyclase; CR3, complement receptor 3; LUV, large unilamellar vesicle.

The acylated segments of homologous RTX toxins (Fig. S1) harbor several conserved aromatic residues, with the homologs of the W876 residue of CyaA (Fig. 1*A*) being located near the first of the two acylated lysine residues (*e.g.*, K860 of CyaA). Based on the recently solved structure of the RTX751 fragment of CyaA in complex with its CR3 receptor (13), we reasoned that the indole ring of the conserved tryptophan 876 might play a structural role in the acylated segment (Fig. 1, *B* and *C*). Therefore, we used CD spectroscopy to examine the impact of a W876L substitution on the overall structure of the acylated segment. To simplify the interpretation of the CD measurements, we used a truncated RTX719 protein comprising the acylated segment fused to a minimal RTX domain with C-terminal capping structure that supports the calcium-dependent vectorial folding of the RTX domain (Fig. 1*D*) (28, 37). As shown in Figure 1*E*, the far-UV CD spectra of the RTX719 W876L protein refolded in the presence of 2 mM calcium ions were indistinguishable from those of the intact RTX719 protein and exhibited a negative peak at 218 nm, which is characteristic for the RTX β-roll structure. This indicated that the W876L substitution did not detectably affect the overall calcium-induced fold of the acylated segment and of the C-proximal RTX structures of CyaA. Therefore, we examined the acylation status and cytotoxic activity of a set of W876F, W876Y, and W876L toxin variants. As shown in Table 1, the co-expressed CyaC acyltransferase modified the CyaA variants to the same extent as the intact toxin, attaching predominantly C16:0 and C16:1 fatty acyl chains to the K860 and K983 residues. Moreover, the ability of the full-length toxin variants to bind CR3-expressing cells, such as THP-1 monocytes or chinese hamster ovary (CHO)-CR3 transfectants, remained largely intact (Fig. 2*A*). The W876L/F/Y substitutions only slightly reduced (∼20%) the specific capacity of the toxin variants to deliver the AC domain and elevate cAMP concentration in CHO-CR3 transfectants that bear high amounts of the toxin receptor on cell surface. Hence, the bulky indole side chain group of W876 could be substituted by smaller aliphatic or aromatic side chains without any major impact on the acylation status and capacity of CyaA to penetrate CR3^+^ leukocytes. However, the substitutions had larger impact on toxin activity on nonactivated THP-1 cells that express low amounts of CR3 (38), and the CyaA W876L/F/Y toxin variants exhibited a strongly reduced capacity to bind and penetrate the membrane of cells lacking CR3, such as mock CHO cells and erythrocytes. (Fig. 2*A*). The reduced erythrocyte binding then strongly reduced the hemolytic capacity (Fig. 2*B*) that is a higher order function (Hill No. > 3) of the number of membrane-inserted toxin molecules oligomerizing into a cation-selective pore (10, 33, 39). As verified for CyaA W876L, the low hemolytic activity was due to a strongly decreased membrane permeabilization activity (Fig. 2*C*). However, once formed, the CyaA W876L pores exhibited similar mean unit conductance and lifetime as pores formed by intact CyaA (Fig. 2, *D*–*F*), showing that the overall structure of the toxin pore was unaffected. In addition, CyaA W876L toxin bound less efficiently to the membrane of large unilamellar vesicles made of phosphatidylcholine (Fig. 2*G*). Overall, these results indicate that the W876L substitution affected the ability of CyaA to penetrate the lipid bilayer of membranes devoid of the CR3 receptor, which decreased the frequency of formation of oligomeric toxin pores. Replacement of the indole group of W876 by aromatic or aliphatic hydrophobic side chains thus strongly affected CyaA activities on cells devoid of CR3.

**Table 1:** Acylation status of CyaA and HlyA variants

protein^a^	modification	K860^b^	K983^b^
CyaA	nonmodified	4	2
	C16:0	20	29
	C16:1	71	55
	C18:1	3	11
CyaA W876L	nonmodified	13	1
	C16:0	14	25
	C16:1	70	64
	C18:1	2	8
CyaA W876F	nonmodified	0	2
	C16:0	35	37
	C16:1	59	53
	C18:1	5	7
CyaA W876Y	nonmodified	1	3
	C16:0	35	36
	C16:1	58	53
	C18:1	6	6
CyaA P848G	nonmodified	13	0
	C16:0	41	32
	C16:1	46	62
	C18:1	0	6


	Modification	K564^b^	K690^b^
HlyA	nonmodified	0	0
	C14:0	44	65
	C14:OH	51	29
HlyA W579L	nonmodified	0	0
	C14:0	49	67
	C14:OH	47	27

a Proteins were produced in *Escherichia coli* strain XL-1 Blue and purified from urea extracts close to homogeneity as described in Experimental procedures.

b Percentage distribution of fatty acid modification of the ε-amino groups of residues K860 and K983 of the CyaA constructs and K564 and K690 of the HlyA constructs. Average values from the analysis of two independent preparations of the CyaA or HlyA variants were shown. The remaining lysine residues of CyaA variants to 100% are acylated with C14:0. The remaining lysine residues of HlyA variants to 100% are acylated with C12:0, C12:0-OH, C14:1, and C14:1-OH.

### Substitution of W579 impairs cytotoxicity of HlyA on β_2_ integrin–negative cells

**Figure 3:**
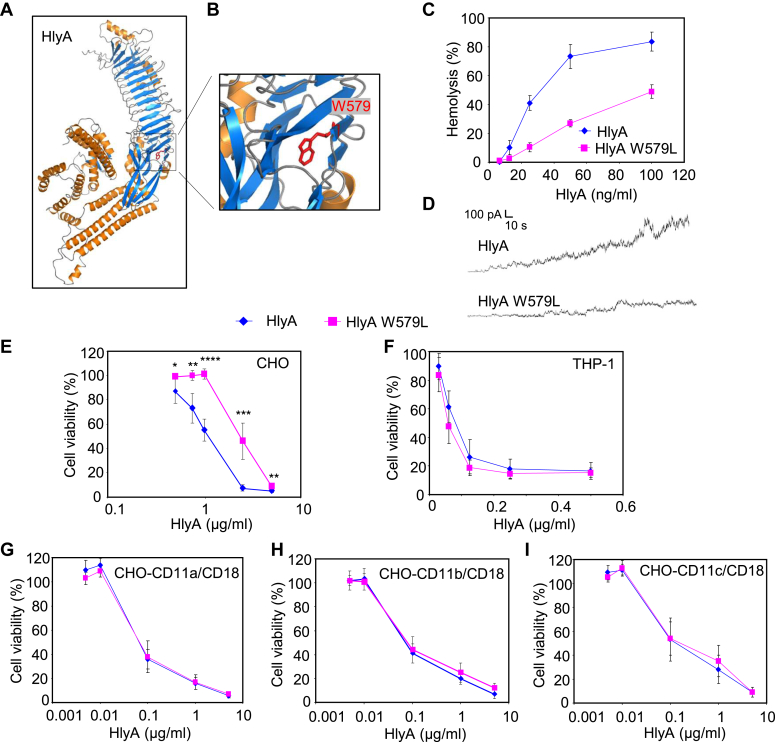
**Substitution of W579 selectively impairs the cytotoxic activity of HlyA on cells not expressing β**_**2**_**integrins.***A*, PyMol view of the most probable model of HlyA (UniProtKB P08715) predicted by AlphaFold. The W579 side chain is highlighted in *red*. *B*, close-up view of the relevant part of the acylated segment of HlyA. *C*, sheep erythrocytes (5 × 10^8^/ml) were incubated at 37 °C with indicated concentration of HlyA, and hemolytic activity was measured after 15 min as A_541nm_. *D*, overall membrane activities of 1.5 nM HlyA variants on asolectin black lipid membranes bathing in 50 mM KCl, 10 mM Tris–HCl (pH 7.4), 2 mM CaCl_2_ under 50 mV applied voltage at 25 °C. The recording was filtered at 10 Hz. Cell viability of HlyA-treated (*E*) mock-transfected CHO cells (1.5 × 10^5^/well), (*F*) THP-1 cells (1.5 × 10^5^/well), (*G*) CHO cells transfected with CD11a/CD18 (1.5 × 10^5^/well), (*H*) CHO cells transfected with CD11b/CD18 (1.5 × 10^5^/well), and (*I*) CHO cells transfected with CD11c/CD18 (1.5 × 10^5^/well), respectively, was determined as the capacity of mitochondrial dehydrogenases to reduce the tetrazolium salt WST-1 to its formazan product after 2 h of exposure to toxin at 37 °C. Each point represents the mean ± SD of at least four independent determinations performed in triplicate with two independent HlyA preparations. Significance of differences was determined using the unpaired Student's *t* test. ∗∗∗∗*p* < 0.0001, ∗∗∗*p* < 0.001, ∗∗*p* < 0.01, ∗*p* < 0.05. HlyA, α-hemolysin.

We next tested whether the corresponding conserved W579 residue of HlyA, located near the first acylation site at K564 (Figs. 1*A* and 3, *A* and *B*), plays a similarly important role in the cytotoxic activity of HlyA. As shown in Table 1, the full-length HlyA W579L toxin was modified by C14:0 and C14:OH acyl chains at residues K564 and K690, like intact HlyA. However, despite similar conductance and lifetime of HlyA and HlyA W579L pores (Fig. S2), the W579L substitution strongly reduced the overall pore-forming activity of HlyA W579L on erythrocytes (Fig. 3*C*) and artificial planar lipid membranes (Fig. 3*D*). Moreover, the cytotoxic activity of HlyA W576L towards mock-transfected CHO cells was significantly lower than that of intact HlyA (Fig. 3*E*). In contrast, the cytotoxic activity of HlyA W579L on β_2_ integrin–expressing human THP-1 cells was the same as that of intact HlyA (Fig. 3*F*). To confirm that the full cytotoxic activity of the HlyA W579L variant was due to the interaction with the β_2_-subunit (CD18) of β_2_ integrins, we tested the cytotoxicity of HlyA W579L on CHO cells expressing three different human β_2_ integrins: CD11a/CD18, CD11b/CD18, and CD11c/CD18, respectively. As shown in Figure 3, *G*–*I*, the HlyA and HlyA W579L proteins were comparably cytotoxic on all three β_2_ integrin–expressing CHO transfectants over a range of toxin concentrations. These cells were much more sensitive to the action of HlyA and HlyA W579L toxins than mock-transfected CHO cells, confirming that cell surface expression of the shared β_2_ integrin subunit enabled high HlyA cytotoxicity (40). Hence, a single W579L substitution impaired the hemolytic, pore-forming, and cytotoxic capacity of HlyA on erythrocytes, artificial membranes, or mock-transfected CHO cells lacking β_2_ integrins, whereas the HlyA W579L variant was fully cytotoxic on cells expressing three different β_2_ integrins.

### Removal of the indole group of W876 increases the thermal stability of CyaA

**Figure 4:**
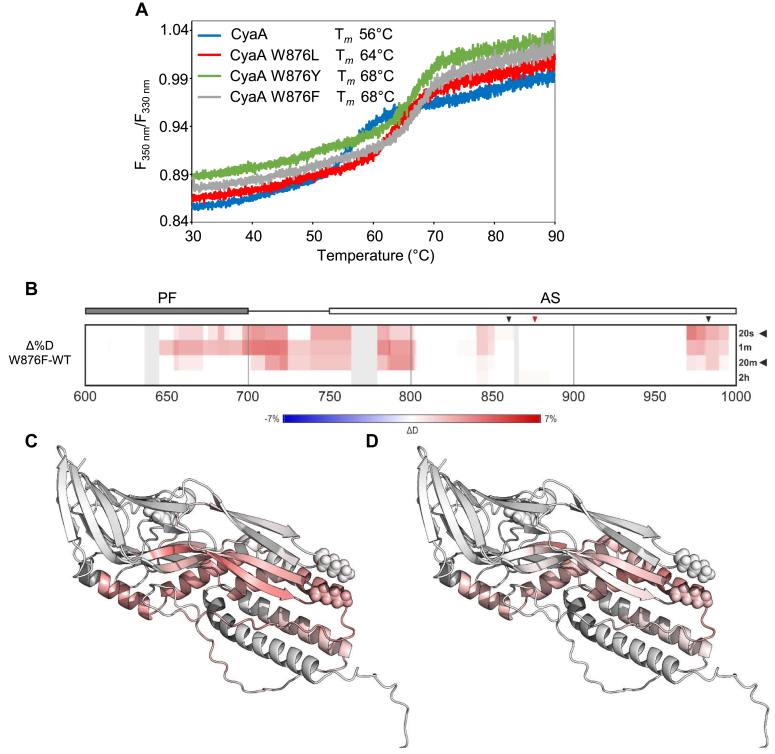
**W876 substitutions increase the thermal stability of CyaA but locally enhance the accessibility of hydrophobic and acylated segments to deuteration.***A*, representative thermal melting curves of refolded calcium-loaded CyaA variants assessed by nanoDSF. The ratios of fluorescence intensities at 350 nm and 330 nm (F350 nm/F330 nm) after heating of the protein samples (0.1 mg/ml) diluted in Tris 50 mM (pH 8.0), NaCl 150 mM, CaCl_2_ 2 mM were followed as a function of temperature. *B*, the difference in deuteration levels between CyaA WT and W876F shown as a differential heat map. Deuteration level of the WT protein was subtracted from that of the W876F mutant. Changes in deuteration are visualized by the *blue* (protection/lower deuteration)-*white* (no change)-*red* (deprotection/higher deuteration) gradient. Domains are depicted above the heat map as well as the positions of acylation (*black arrowheads*) and the W876F site (*red arrowhead*). Regions in *gray* were not covered by the HDX-MS data. Two exchange time points, 20 s (*C*) and 20 min (*D*) indicated by the *arrowheads* on the *right* side, are visualized on the model of CyaA (residues 600–1000) using the same color gradient. Heat map covering the entire CyaA sequence is provided in the Supporting information - Fig. S3. AS, acylated segment; HDX-MS, Hydrogen/deuterium exchange followed by mass spectrometry; nanoDSF, nanodifferential scanning fluorimetry; PF, pore-forming domain.

When the thermal unfolding of the full-length calcium-loaded CyaA toxin variants was analyzed by nanodifferential scanning fluorimetry (nanoDSF), the CyaA variants exhibited importantly increased thermal stabilities compared to intact CyaA (Fig. 4*A* and Table 2). Replacement of the W876 indole by smaller aliphatic or aromatic groups yielded average T_*m*_ values that were 4 °C (W876L), 7.3 °C (W876Y), and 8 °C (W876F) higher, respectively (from 59.0 °C up to 67.0 °C, Table 2).

**Table 2:** Melting temperature values of CyaA variants

protein^a^	T_*m*_ (°C)^b^
CyaA	59.0 ± 2.0
CyaA W876L	63.0 ± 1.0
CyaA W876F	67.0 ± 1.1
CyaA W876Y	66.3 ± 2.8
CyaA W876Q	59.5 ± 1.5
CyaA W876F+V822M	62.8 ± 2.7
CyaA P848G	61.5 ± 1.2

a Proteins were produced in *Escherichia coli* strain XL-1 Blue and purified as described Experimental procedures.

b Melting temperature (T_*m*_) values, corresponding to the inflection points of the unfolding curves, were performer by nanoDSF. Average T_*m*_ values ± SD from analysis of at least two independent toxin preparations were shown. N = 4 to 11.

### HDX-MS reveals structural changes in the hydrophobic and acylated segment of CyaA W876F

To gain more insight into impact of the W876F mutation on CyaA structure, we performed HDX-MS experiments to characterize the folding dynamics of individual toxin segments over time in the presence of 4 mM free Ca^2+^ ions. The overall deuteration profile corresponded well with the structuring of CyaA. No significant differences in deuteration kinetics were observed between the CyaA W876F and WT toxins for the AC domain, the ‘AC-to-Hly’ linker segment, the N-terminal portion of the hydrophobic pore-forming domain, and the calcium-binding RTX domain, respectively, showing that the folding and structure of these regions were not affected by the W876F substitution (Figs. S3 and S4, panels A–G). In contrast, importantly increased deuterium uptake was observed for the W876F toxin variant in the structured regions located at the interface between the hydrophobic domain and the acylated segment, delimited by residues ∼650 to 760 and ∼780 to 800 (Fig. 4, panels B, C and D and Figs. S3 and S4). In addition, residue stretches ∼841 to 851 and ∼970 to 995 of the two long loops protruding from the β-roll core of the acylated segment (with the acylated K860 and K983 residues at their tips) were also importantly more accessible to deuteration in the CyaA W876F variant than in WT CyaA (Fig 4, panels B, C, D and Figs. S3 and S4). Hence, the substitution of the conserved W876 residue selectively affected the deuterium uptake accessibility of the interface between the hydrophobic and acylated segment and the interaction surface of the two loops exposing the acylated K860 and K983 residues.

### The bulky indole group of the W876 plays a key functional role in CyaA structure

**Figure 5:**
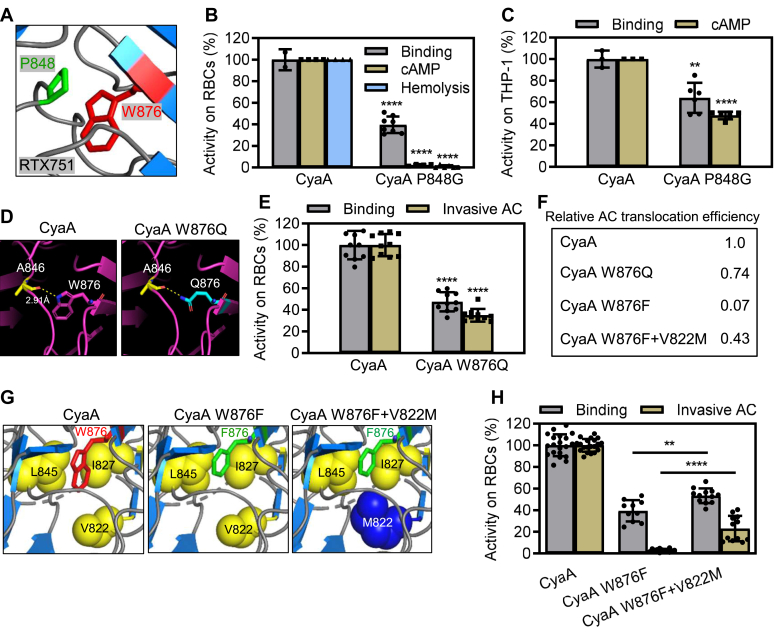
**The indole group of the W876 residue plays a functional role in CyaA structure.***A*, PyMol view of the portion of the acylated segment of RTX751 (PDB 7USL). The W876 and P848 residues are rendered in *red* and *green*, respectively. *B*, cell binding to erythrocytes was determined using the AC assay as described in detail in panel 2A. AC domain translocation was assessed by determining intracellular cAMP concentration in erythrocytes as described in Experimental procedures. Hemolytic activity was measured after 3 h as in Figure 2*B*. *C*, cell binding and intracellular cAMP concentrations were determined using THP-1 cells as in Figure 2*A*. *D*, PyMol view of portions of the acylated segment of CyaA (PDB 7USL, left panel). The side chains of W876, Q876, and A846 are represented by sticks, with *red* oxygen atoms of backbone carbonyl and blue nitrogen atoms of W876 or Q876 side chains. The *yellow dashed line* depicts a hydrogen bond. *E* and *H*, sheep erythrocytes (5 × 10^8^/ml) were incubated with CyaA proteins (1 μg/ml) in the presence of 2 mM CaCl_2_ at 37 °C for 30 min before cell binding, and invasive AC activity was determined using the AC assay. The AC domain translocation efficiency factor in panel (*F*) is defined as the ratio of relative cell-invasive AC activity to relative cell-binding capacity, both determined as the amount of cell-bound AC enzyme ± digestion of extracellular AC enzyme by added trypsin. *G*, PyMol view of portions of the acylated segment of CyaA (PDB code 7USL, *left* panel). The side chain of substituting M822 residue is depicted as *blue spheres*. Residue V822 is represented by *yellow spheres*. The side chains of W876 and F876 are depicted as *red* and *green sticks*, respectively. Activities of CyaA variants are expressed as a percentage of intact CyaA activity and represent means ± SD of at least five independent determinations performed in duplicate with two different toxin preparations. Significance of differences was tested using one-way ANOVA. ∗∗∗∗*p* < 0.0001, ∗∗*p* < 0.01. AC, adenylyl cyclase; RTX, Repeats in ToXins.

Examination of the tertiary structure of the RTX751 fragment of CyaA (13) revealed a hydrophobic interaction between the binuclear aromatic indole ring of tryptophan 876 and the parallel pyrrolidine of proline 848 (Fig. 5*A*). To estimate the interaction strength between the W876 and P848 side chains, we performed quantum-mechanical calculations on three models (Fig. S5), using the density functional theory with an empirical dispersion correction (DFT-D) and the *ab initio* MP2 method with a large aug-cc-pVDZ basis set. The calculated interaction energies (Table 3) showed similar trends in all three models, and both DFT-D and MP2 predicted high interaction strengths of over 5 kcal/mol. We thus tested the functional role of this interaction by a P848G substitution. As documented in Figure 5*B*, despite a cell-binding capacity of ∼40%, the CyaA P848G protein exhibited almost nil AC translocation and hemolytic activities on erythrocytes that do not express CR3. In striking contrast, the binding of CyaA P848G to CR3-expressing THP-1 cells was reduced by only ∼35%, while the CyaA P848G toxin retained ∼50% of the specific AC delivery capacity of intact CyaA (Fig. 5*C*). Intriguingly, the CyaA P848G T_*m*_ value of 61.5 °C was only 2.5 °C higher than that of intact CyaA (Table 2).

**Table 3:** Interaction energy (in kcal/mol) calculated for the W876-P848 pair in various models using two different QM methods

Method	“Trp-pro” model	“Large” model	“Small” model
DFT-D/TPSS/TZVP	−6.0	−7.5	−5.1
MP2/aug-cc-pVDZ	−5.9	−7.3	−5.1

Abbreviation: QM, quantum-mechanical.

Aside from the capacity to fill hydrophobic cavities, the tryptophan indole group can also facilitate solvation of folded proteins through its NH group (41). To evaluate the role of the W876 indole NH group, we replaced this residue with a polar glutamine residue which positions an NH group at a similar distance from the peptide backbone (Fig. 5*D*). As shown in Fig. S6, the ability of the CyaA W876Q toxin to penetrate CR3-expressing THP-1 monocytes remained intact and the W876Q substitution only caused a two-fold reduction of the specific erythrocyte-binding capacity of CyaA W876Q compared to intact CyaA (Fig. 5*E*), whereas its cell-invasive activity was only minimally affected (∼74% of intact CyaA, Fig. 5*F*). Also, the T_*m*_ value of CyaA W876Q remained close to that of intact CyaA (59.0 °C for CyaA *versus* 59.5 °C for CyaA W876Q, Table 2). This contrasts with the impact of the W876F/Y/L substitutions that strongly increased the T_*m*_ of the toxin variants and ablated their capacity to deliver the AC domain across erythrocyte membrane devoid of CR3 (*cf.* Figs. 2*A* and 4*C*). The presence of a polar uncharged NH group at the tip of the side chain at position 876 thus plays a key functional role in CyaA penetration of cellular membranes lacking CR3.

Since the CyaA W876F variant exhibited ∼10-fold lower AC-translocation efficiency, defined as the ratio of relative cell-invasive AC activity to relative cell-binding capacity, exhibiting importantly higher thermostability at the same time than CyaA W876Q, (Fig. 5*F* and Table 2), we reasoned that the bulky tryptophan side chain at position 876 may destabilize the toxin structure to facilitate its membrane penetration. To test this hypothesis, the stabilizing W876F substitution was combined with a secondary cavity-filling V822M substitution predicted to destabilize the structure (Fig. 5*G*). The resulting CyaA W876F+V822M toxin variant not only bound and intoxicated CR3-expressing THP-1 monocytes like intact CyaA (Fig. S6) but addition of the V822M substitution partly rescued the erythrocyte membrane binding and AC translocation defect of the CyaA W876F variant (Fig. 5, *H* and *F*). Moreover, the T_*m*_ of the double variant CyaA W876F+V822M toxin was less elevated, suggesting that the V822M substitution destabilized it relative to CyaA W876F (62.8 °C *versus* 68 °C, Table 2). These results suggest that the hydrophobic interactions and local structure-destabilizing and T_*m*_-decreasing effects of the bulky indole group of the W876 residue are required for membrane insertion and AC translocation of CyaA on CR3-deficient cells.

## Discussion

**Figure 6:**
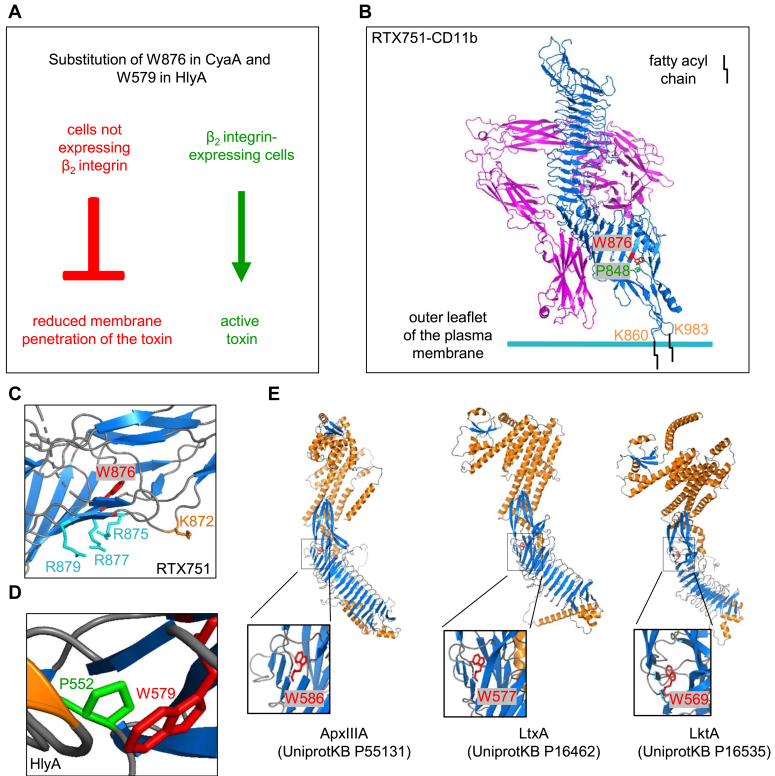
**Conserved tryptophan residues in the acylated segments of RTX toxins.***A*, summary of the role of the conserved tryptophan of the acylated segment in the action of CyaA and HlyA. *B*, the stacking-like interaction between the binuclear aromatic indole ring of W876 and pyrrolidine ring of P848 residue side chains stabilizes the positioning of the loop bearing on its tip the palmitoylated K860 residue. Schematic representation of the CR3-bound RTX751 fragment of CyaA (PDB 7USL), depicting the orientation of the K860 and K983-linked palmitoyl acyl chains (*black*) pointed to the plane of the membrane and mediating the first contact of the acylated segment of CyaA (in *blue*), with the outer leaflet of the lipid bilayer of cell membrane. The CD11b subunit of CR3 is labeled in *magenta*, the W876 residue is in *red*, P848 in *green*, and K860 and K983 residues are in *orange*. *C*, PyMol view of the corresponding RTX751 portion (PDB 7USL). Tryptophan 876 is in *red*, lysine 872 in *orange*, and the arginine residues 875, 877, and 879 are in *cyan*. *D*, PyMol view of the AlphaFold-predicted structure of the acylated segment portion of HlyA predicted by (UniProtKB P08715). The conserved tryptophan W579 of HlyA is indicated by *red* color, and proline 552 is in *green*. *E*, AlphaFold models of *Actinobacillus pleuropneumoniae* ApxIIIA, *Aggregatibacter actinomycetemcomitans* LtxA, and *Mannheimia haemolytica* LktA toxins. The conserved tryptophan residues are highlighted in *red* in the zoomed-out image. CR3, complement receptor 3; HlyA, α-hemolysin; RTX, Repeats in ToXins.

We propose that conserved tryptophan residues in the vicinity of the first acylated lysine residues (*e.g.*, W876 near K860 of CyaA and W579 near K564 of HlyA) determine the interaction and structural configuration of the two acylated loops protruding from the acylated segments. Interaction of these loops would then be specifically required for β_2_ integrin receptor–independent (nonassisted) membrane penetration of these prototypical RTX toxins (Fig. 6*A*). In contrast, the interaction and cooperation of the acylated loops is not necessary when the toxin is docked by β_2_ integrin receptors in a defined orientation onto cell surface. We speculate that receptor binding positions the toxin in an orientation that favors insertion of the lysine-attached acyl residues into the outer leaflet of the cell membrane (13), and this mediates subsequent toxin penetration into the lipid bilayer. In the absence of β_2_ integrins on cell surface, toxin molecules are loosely adsorbed onto glycans of membrane glycolipids and glycoproteins (22, 23, 24, 42), likely in a variety of orientations relative to the plane of cell membrane. The toxin molecules thus need to tumble in a glycan association-dissociation process and stochastically explore numerous positions relative to membrane surface, until a configuration leading to membrane penetration is reached.

The results of accessibility to deuteration of CyaA W876F protein segments suggest that interactions of the indole group of W876 with residues occupying the hydrophobic core of the acylated segment of CyaA rule the positioning of the hydrophobic segments of CyaA, as well as the interaction of the loops bearing the amide-linked palmitoyl chains on the K860 and K983 residues at their tips (13). The enhanced deuteration of the interface between these acylated loops in the W876F toxin variant (*cf.* Fig. 4, *B*–*D*) reveals a relaxation of their interaction through hydrogen bonding (43). It is plausible to speculate that loosening of the interaction between the acylated loops accounted for the selective defect of CyaA W876F toxin variant in penetration of membranes lacking the toxin-positioning β_2_ integrin receptor. As also schematically depicted in Figure 6*B*, the two fatty acyl chains on residues K860 and K983 likely mediate the first contact of the CyaA molecule with the outer leaflet of the membrane lipid bilayer, irreversibly anchoring the protein into cell membrane. When positioned on cell surface in a CR3-assisted manner, membrane insertion would be facilitated by orienting of the acylated loops towards the membrane plane (13). Intriguingly, the single palmitoyl residue linked to the K983 residue appears to be necessary and sufficient for CyaA penetration across the cellular membrane, as the CyaA K860R variant monoacylated on the K983 residue exhibits a full capacity to translocate the AC domain into cytosol of CR3-expressing cells, such as mouse J774A.1 macrophages (44). Moreover, intact CyaA monoacylated selectively on the K983 residue (by an A140V variant of the CyaC acyltransferase) possessed a capacity to penetrate CR3^−^ erythrocyte membrane that was proportional to the extent of K983 residue acylation (45). In contrast, monoacylation of the K860 residue in the CyaA K983R mutant yielded only a 5- to 6-times reduced (∼15%) specific CR3^+^ membrane translocation capacity, albeit the K983R toxin variant monoacylated on K860 still exhibited a full capacity to tightly associate with a CR3^+^ cell membrane. Hence, membrane insertion of the palmitoyl chain attached to the K860 residue was sufficient for anchoring of CyaA into the CR3^+^ membrane but was largely inefficient in the initiation of toxin translocation across the CR3^+^ membrane. In line with that, the capacity of the CyaA K983R toxin to tightly associate with CR3^−^ erythrocyte membrane was about 10-fold reduced and its capacity to translocate the AC domain across CR3^−^ erythrocyte membrane was close to nil, around ∼1% of that of intact CyaA (44). The sum of these observations suggests that the acyl linked to K860 residue cannot functionally replace the K983-linked acyl and that membrane translocation of CyaA needs to be initiated by insertion of the K983-linked acyl into cell membrane. It is tempting to speculate that this essential toxin activity step is selectively perturbed in the CyaA W876F toxin variant when this is not docked on cell surface and positioned relative to cell membrane plane through an interaction with CR3. A plausible hypothesis would be that in the absence of CR3, the loop bearing acylated K860 residue must interact with the loop bearing the essential acyl on K983 to stabilize its functional positioning relative to the rest of the toxin molecule. Once this interaction is perturbed in the CyaA W876F toxin variant, the essential step of membrane insertion of the acyl linked to K983 would be hindered by its enhanced mobility relative to the rest of the CyaA molecule. Hence, in the absence of CR3 receptor-mediated docking, a futile initial insertion of the K860-linked acyl into the CR3^−^ membrane may be favored.

The selective loss of CyaA W876F/Y/L toxin activity on CR3^−^ cells suggests that the local bulge of the structure in the vicinity of the acylated K860 residue plays a role in toxin interaction with CR3-free membranes. Maintaining this local structure by the bulky indole group of W876 can be functionally important for CR3-independent membrane insertion of CyaA. Notably, the sequence around the W876 residue consists of a cluster of positively charged residues _872_**K**QD**R**W**R**I**R**_879_ (*cf.* Fig. 1). It is tempting to speculate that by destabilizing the packing of the local structure, the presence of the indole group of W876 enhances the mobility of the surrounding positively charged side chains of the arginine residues 875, 877, and 879 (Fig. 6*C*), thus enabling their interaction with the negatively charged sialylated gangliosides on the membrane surface (22, 23) or even enable their insertion in-between the negatively charged phospholipid headgroups. This would plausibly be expected to alter the local packing of lipids in the outer leaflet of the lipid bilayer and may facilitate the insertion of the K860-linked palmitoyl residue into the membrane. In contrast, upon CR3-dependent docking of CyaA with the acyl chains brought in proximity and pointed to the plane of the membrane (Fig. 6*B*), the toxin-positioning effect would primarily allow the exposure and membrane insertion of the aliphatic palmitoyl chain sticking out from the tip of the K983-containing linker loop. Whether the indole group of W876 is present or not, this would be sufficient for a conformational change of the toxin molecule, triggering an irreversible insertion of its hydrophobic and amphipathic segments into cell membrane and leading to translocation of the N-terminal AC domain across the membrane into cell cytosol.

In the solved (RTX751) or AlphaFold-modeled structures of CyaA and HlyA (*cf.* Figs. 1, *B* and *C* and 3, *A* and *B*), the indole groups of W876 and W579 residues are buried in the hydrophobic cores of the β-roll structure of the acylated segments and are thus unlikely to directly interact with the lipid bilayer of target cell membranes during toxin insertion. Intriguingly, the CyaA W876L/F/Y variants, having the indole side chain at position 876 replaced by aromatic or aliphatic side chains, exhibited increased thermal stability. Paradoxically, these more stable CyaA W876L/F/Y toxin variants had strongly reduced capacity to penetrate the membrane of cells lacking the CyaA receptor CR3 but retained almost full activity on CHO-CR3 cells expressing high CR3 receptor amounts (*cf.* Fig. 2*A*). Similarly, the analogous HlyA W579L variant was fully active on β_2_ integrin–expressing cells, while its cytotoxic and cytolytic activities on cells lacking β_2_ integrins were heavily impaired (*cf.* Fig. 3). It thus seems unlikely that the indole group of W876 of CyaA or that of W579 of HlyA plays any crucial role in the early folding steps of the acylated segments or determines the overall structure of these RTX toxins. Intriguingly, the defect in membrane penetration of CyaA W876F on erythrocytes could be partially rescued by additional introduction of a cavity-filling V822M substitution, which reduced the thermal stability of the protein to a value closer to that of intact CyaA. When combined with the aromatic W876F substitution, the S-methyl thioether side chain of methionine introduced at position 822 mimicked in part the functionally important hydrophobic cavity-inflating effect of the bulky indole group of W876.

### The CyaA variants analyzed here reveal additional contributions of the indole group to toxin function

First, the indole nitrogen forms a hydrogen bond with the carbonyl of the main chain of the A846 residue of CyaA. This interaction over 2.91 Å appears to be important for proper membrane translocation of CyaA on cells lacking CR3. The W876F substitution selectively debilitated this activity, whereas replacement of the indole group by a glutamine chain, bearing a nitrogen at a distance enabling an interaction with the carbonyl of A846, yielded a CyaA W876Q toxin with largely preserved membrane penetration activity on erythrocytes lacking CR3.

Second, the indole group packs onto the pyrrolidine of the P848 residue. Quantum-mechanical calculations of the strength of this interaction suggested an energic contribution of over 5 kcal/mol, which is similar to high values calculated earlier for the Trp-Pro interaction in a “stack-like” arrangement (46). The P848G substitution had little effect on the thermal stability of the CyaA but yielded a near-complete loss of CyaA P848G toxin activity on CR3^−^ erythrocytes. At the same time, the cell-penetrating activity of the P848G toxin on CR3-expressing monocytes was largely preserved. These data suggest that interaction between the W876 and P848 residue side chains stabilizes the positioning of the loop acylated on K860 residue and its interaction with the other loop, thereby stabilizing the position of the palmitoylated K983 residue and making it available for insertion into CR3^−^ cell membrane (Fig. 6*B*, (13)). Indeed, replacement of P848 by glycine is expected to affect the folding of the acylated segment and enhance the flexibility of the main chain due to loss of the packing of the pyrrolidine onto W876. As deduced from an AlphaFold model, a similar arrangement of W579 and P552 would be present in the acylated segment of HlyA (shown in Fig. 6*D*). This suggests a role of the tryptophan–proline interaction also in the mechanism of β_2_ integrin–independent membrane penetration of HlyA. As further inferred form the AlphaFold models of other RTX toxins targeting β_2_ integrins, such as LtxA, LktA, or ApxIIIA (19), all these toxins appear to bear a conserved tryptophan with the indole group oriented into the hydrophobic core of the acylated segment (Fig. 6*E*). This suggests that these conserved tryptophan residues of the acylated segments likely play a similar role also in other pore-forming RTX family leukotoxins.

## Experimental procedures

### Plasmid construction

The plasmid pT7CACT1 was used to produce CyaC-activated CyaA (47). Plasmid pT7hlyC-hlyA was used to produce HlyC-activated HlyA with a C-terminal double 6xHis tag (48). The expression vector encoding the RTX719 protein was derived from pT7CT7ACT1-ΔNdeI (28, 49). Oligonucleotide-directed PCR mutagenesis was performed to construct the following: (i) pT7CACT1-derived plasmid for the construction of CyaC-activated CyaA W876L, CyaA W876Y, CyaA W876F, CyaA W876Q, CyaA W876F+V822M, and CyaA P848G; (ii) pT7hlyC-hlyA–derived plasmid for the construction of HlyC-activated HlyA W579L; and (iii) mutation W876L was introduced into the *CyaA* fragment of pT7CT7-RTX719 by site-directed PCR mutagenesis.

### Production and purification of CyaA, HlyA, and RTX719

CyaA and HlyA variants were produced in *E. coli* XL-1 Blue (Stratagene). The CyaA, HlyA, and RTX719 variants were purified as described earlier (7, 28, 30). For CD analysis, imidazole was removed from the samples on Amicon Ultra 10k (Merck). Protein purity was checked by SDS-PAGE (Fig. S7), and protein concentrations were determined by Bradford assay (Bio-Rad).

### Cell lines

Human monocytes THP-1 (ATTC number TIB-202) were cultured in RPMI medium supplemented with 10% heat-inactivated fetal calf serum (FCS) and antibiotic antifungal solution. CHO cells (CHO-K1, ATCC CCL-61) stably transfected with human CD11a/CD18, CD11b/CD18, CD11c/CD18, or mock-transfected were prepared previously (21) and grown in F12K medium (GIBCO Invitrogen) supplemented with 10% FCS and antibiotic antifungal solution. Prior to assays, RPMI/F12K was replaced with Dulbecco’s modified Eagle’s medium (D-MEM, 1.9 mM Ca^2+^) without FCS.

### Determination of AC activity

Enzymatic AC activity was measured in the presence of calmodulin (1 μM) as described previously (50). One unit of AC corresponds to 1 μmol cAMP formed in 1 min at pH 8 at 30 °C.

### Binding and cell-invasive activities of CyaA on sheep RBC

Sheep red blood cells (RBCs, LabMediaServis) were washed in TNC buffer (50 mM Tris–HCl at pH 7.4, 150 mM NaCl, 2 mM CaCl_2_), adjusted to 5 × 10^8^ cells/ml, and incubated with CyaA (1 μg/ml) for 30 min at 37 °C in TNC buffer. Erythrocyte binding was measured by determining the membrane-associated AC activity as previously described (30). Cell invasive AC was measured by determining the AC protected against externally added trypsin upon internalization into sheep erythrocytes as previously described (28, 30). The AC domain translocation efficiency factor (*cf.* Fig. 5*F*) is defined as the ratio of relative cell-invasive AC activity to relative cell-binding capacity, both determined as the amount of cell-bound AC enzyme ± digestion of extracellular AC enzyme by added trypsin.

### Binding of CyaA to nucleated cells

THP-1, CHO, and CHO-CD11b/CD18 cells (10^6^/ml) were incubated in D-MEM with CyaA (1 μg/ml) for 30 min at 4 °C, and membrane-associated AC enzyme activity was determined using AC assay (28).

### Binding of CyaA to large unilamellar liposomes

Large unilamellar vesicles prepared from L-α-phosphatidylcholine (type II-S, Sigma) were prepared by extrusion of multilamellar hand-shaken liposome vesicles in TNC buffer, using LiposoFast Basic apparatus (Avestin) with a polycarbonate membrane of 100 nm pore diameter (Avestin). CyaA (1 μg/ml) was incubated with liposome suspensions containing 1 mg of lipids in TNC. After 15 min of incubation at 37 °C, the liposomes were washed twice in TNC and once in 0.1 M Na_2_CO_3_ pH 10.5. For each washing step, the liposome suspensions were pelleted at 51,000*g* for 30 min at 4 °C and resuspended in the indicated buffers (51). The membrane-associated AC enzyme activity was determined using AC assay.

### cAMP determination

AC translocation was assessed by determining the intracellular cAMP concentration after incubation of THP-1, CHO CD11b/CD18, and CHO cells (1.5 × 10^5^/well in D-MEM medium) or RBC (5 × 10^8^/ml in modified Hanks' balanced salt solution: 140 mM NaCl, 5 mM KCl, 2 mM CaCl_2_, 3 mM MgCl_2_, 10 mM Hepes–Na, 50 mM glucose, pH 7.4) for 30 min at 37 °C with various concentrations of CyaA within the linear range of the dose-response curve (250, 125, 62.5, 31.25, and 15.6 ng/ml for THP-1 cells; 50, 25, 12.5, and 6.25 ng/ml for CHO CD11b/CD18 cells; 10, 5, 2.5, 1.25, 0.62, and 0.31 μg/ml for CHO cells; 1 μg/ml and 2 μg/ml for RBC). The cAMP was measured by competitive immunoassay (52).

### Hemolytic activity

Hemolytic activity was measured at 37 °C in TNC buffer as hemoglobin release (A_541_) from washed sheep erythrocytes (5 × 10^8^ cells/ml).

### Cell viability assay

Cell viability was determined in D-MEM using the WST-1 assay kit (Roche).

### LC-MS analysis

The mass spectrometry (MS) analysis was performed using a solariX XR FTMS instrument equipped with a 15 T superconducting magnet and a Dual II ESI/MALDI ion source (Bruker Daltonics), as described earlier (28). Spectra were processed using the Data Analysis 4.4 software package (Bruker Daltonics, https://www.bruker.com/en/services/software-downloads.html), and the extracted data were searched either against the FASTA of an intact toxin molecule (CyaA, UniProtKB P0DKX7; HlyA, UniProtKB: P08715) or its sequence modified in accordance with corresponding mutation using Linx software (RRID:SCR_018657, https://peterslab.org/downloads.php). The acylation status of lysines was determined by comparing the relative intensity ratios between acylated peptide ions and their unmodified counterparts (7).

### Hydrogen/deuterium exchange mass spectrometry

To study the effect of W876F mutation on the structure of CyaA, HDX-MS analyses were performed. The H/D exchange was performed at 21 °C by a PAL DHR robot (CTC Analytics AG) controlled by Chronos software (Axel Semrau, https://software.axelsemrau.de). Protein in 8M urea, 50 mM Tris–HCl pH 8.0, 2 mM EDTA was diluted by 50 mM Hepes pH 7.4, 150 mM NaCl, 4 mM CaCl_2_ buffer to lower the urea concentration to 2.4 M and induce protein folding through the addition of Ca^2+^. Next, the protein was diluted by the Hepes buffer containing 2.4 M urea to a final 5 μM protein concentration. The exchange was started by a 5-fold dilution of this protein solution with a D_2_O-based buffer (50 mM Hepes pH 7.4, 150 mM NaCl, 2.4 M urea, 4 mM CaCl_2_). Four exchange times (20 s, 1 m, 20 m, 2 h) were followed, and two-time points, 20 s and 20 min, were replicated (n = 4 and n = 3, respectively). Exchange was by stopped by 1:1 (vol:vol) mixing of the exchange reaction with the quench buffer (5.6 M urea, 1 M glycine–HCl pH 2.3). Samples were immediately injected onto the LC system consisting of a temperature-controlled box and Agilent Infinity II UPLC (Agilent Technologies) coupled to an ESI source of timsTOF Pro with PASEF (Bruker Daltonics). The LC setup was cooled to 0 °C to minimize the back-exchange and consisted of immobilized pepsin custom-made column (bed volume 66 μl), trap column (SecurityGuard ULTRA Cartridge UHPLC Fully Porous Polar C18, 2.1 mm ID, Phenomenex), and an analytical column (Luna Omega Polar C18, 1.6 μm, 100 Å, 1.0 × 100 mm, Phenomenex). Samples were digested and peptides desalted by 0.4% formic acid (FA) in water delivered by the 1260 Infinity II Quaternary pump at 100 μl.min^−1^. Water-acetonitrile gradient (10%–45%; solvent A: 0.1% FA in water, solvent B: 0.1% FA, 2% water in acetonitrile) followed by a step to 99% B was used to elute and separate the desalted peptides. The solvents were driven by the 1290 Infinity II LC system pumping at 40 μl.min^−1^. Mass spectrometer operated in the MS mode with 1 Hz data acquisition rate and with the tims turned off. The LCMS data were peak picked in DataAnalysis (Bruker Daltonics) and exported to text files. These files, together with the list of identified peptides and sequences of CyaA WT and W876F, were uploaded to a DeutEx software (https://deutex.org) (53) and processed. Data visualization was done using MSTools (http://peterslab.org/MSTools/index.php, (54)) and in PyMol. For peptide identification, the same LC-MS setup was used but the mass spectrometer was operated in data-dependent MS/MS mode with PASEF active. The LC-MS/MS data were searched using MASCOT (v 2.7, Matrix Science) against a custom-built database combining a common cRAP.fasta (https://www.thegpm.org/crap/) and the sequences of CyaA WT, W876F, and pepsin. Search parameters were set as follows: precursor tolerance 10 ppm, fragment ion tolerance 0.05 Da, decoy search enabled, FDR ˂1%, IonScore ˃20, and peptide length ˃5. Variable modifications—Stearoyl, Myristoleyl, Myristoyl-OH, Myristoyl, Steroyl 18:1, Palmitoleyl, Palmitoyl—were set at Lys.

### CD spectroscopy

The far-UV CD spectra were recorded at 25 °C using a Chirascan-plus spectrometer (Applied Photophysics) in rectangular quartz Suprasil cuvettes with 1 mm path length (110-QS, Hellma). Protein samples were diluted in 5 mM Tris–HCl (pH 8.0) and 20 mM NaCl in the presence of 2 mM CaCl_2_ and measured at wavelengths from 200 to 260 nm at a scan speed of 1 nm/s. The spectra of the buffers were subtracted from the protein spectra, and the molar ellipticity was expressed in degrees square centimeter per decimole [deg.cm^2^.dmol^−1^].

### Planar lipid bilayer membrane measurements

Assays on planar lipid bilayers (black lipid membranes) were performed in Teflon cells as described previously (28). The membrane was formed by the painting method using L-α-phosphatidylcholine (type II-S, Sigma-Aldrich) in n-decane–butanol (9:1, vol/vol). Both compartments contained 150 mM KCl, 10 mM Tris–HCl (pH 7.4), and 2 mM CaCl_2_, and the temperature was 25 °C. Membrane current was recorded with Ag/AgCl electrodes (Theta) with salt bridges (applied voltage 50 mV), amplified with LCA-200-100G and LCA-200-10G amplifiers (Femto), and digitized with a LabQuest Mini A/D converter (Vernier). For lifetime determination, dwell times were determined using QuB software (http://go.qub.ac.uk) with 100 Hz low-pass filter. Kernel density estimation was fitted with an exponential function using Gnuplot software (http://www.gnuplot.info).

### Thermal stability

Thermal stability assays were performed by nanoDSF using a Prometheus NT.48 instrument (NanoTemper Technologies). The Ca^2+^-loaded protein samples (0.1 mg/ml) were diluted in 50 mM Tris–HCl (pH 8.0), 150 mM NaCl, and 2 mM CaCl_2_ and loaded into nanoDSF grade standard capillaries (NanoTemper Technologies). The measurements were conducted from 30 to 90 °C (with a temperature ramp of 1.5 °C/min) under constant monitoring of tryptophan fluorescence at 350 and 330 nm. The melting temperature (T_*m*_) values, corresponding to the inflection points of the unfolding curve, were determined by using a PR.ThermControl (NanoTemper Technologies).

### Calculation of interaction energy

The geometric configuration of Trp876 and Pro848 was extracted from the RTX751 structure (PDB code 7USL). In the “Trp-Pro” model, the protein backbone was cut at the peptide bond. In the “large” model, the protein backbone was cut at the C-Cα bond. In the "small" model, only the side chain of tryptophan was considered (starting from the Cβ atom), while proline was represented as a pyrrolidine to preserve its heterocyclic structure (46). The molecular visualization programs Pymol (the PyMOL Molecular Graphics System, Version 2.4 Schrödinger, LLC.), ChimeraX (55), and MOLDEN (https://www3.cmbi.umcn.nl/molden/) were used to create the initial structures. Geometry optimization of added hydrogen atoms was performed using DFT/B3LYP/6-31G∗∗ method. The W876-P848 interaction energy was calculated in each model at two different levels of theory. The DFT-D method with the TPSS exchange, correlation functionals and the D3 version of Grimme’s dispersion correction with the original D3 damping function (56), and the *ab initio* MP2 method with the aug-cc-pVDZ basis set were used. Counterpoise correction for basis set superposition error was applied to all interaction energy calculations. The calculations were performed in Gaussian 16 (57), and MOLDEN was used to analyze the results of the geometry optimizations.

### Structure prediction

AlphaFold (58) was used to predict the structure of RTX toxins using standard settings.

### Statistical analysis

Results were expressed as arithmetic mean ± SD of the mean. Unpaired Student’s *t* test or one-way ANOVA was used to calculate statistical significance (GraphPad Prism 9.1.1; GraphPad Software, https://www.graphpad.com/). Significant differences from intact toxin are indicated by asterisks (∗*p* < 0.05; ∗∗*p* < 0.01; ∗∗∗*p* < 0.001; ∗∗∗∗*p* < 0.0001).

### Data availability

The mass spectrometry proteomics data have been deposited to the ProteomeXchange Consortium (59) *via* the PRIDE (60) partner repository with the data set identifier PXD041207 and PXD041280. The datasets generated during this study are available from the corresponding author on reasonable request.

## Supporting information

This article contains supporting information.

## Conflict of interest

The authors declare that they have no conflict of interest with the contents of this article.
