# Supplementary material for: A conserved tryptophan in the acylated segment of RTX toxins controls their β2 integrin–independent cell penetration
Source: J Biol Chem. 2023 Jun 28;299(8):104978. doi: 10.1016/j.jbc.2023.104978 (PMC10392135; doi:10.1016/j.jbc.2023.104978)
Supplement: Supporting Figures S1–S7 [file mmc1.pdf]

Supporting Information

A conserved tryptophan in the acylated segment of RTX toxins controls their β_2- integrin-independent cell penetration

Adriana Osickova[1,#], Sarka Knoblochova[1,#], Ladislav Bumba[1], Petr Man[1], Zuzana Kalaninova[1,2], Anna Lepesheva[1,2], David Jurnecka[1], Monika Cizkova[1], Lada Biedermannova[3], Jory A. Goldsmith[4], Jennifer A. Maynard[5], Jason S. McLellan[4], Radim Osicka[1], Peter Sebo[1], and Jiri Masin[1,*]

```
CyaA    718IEKLANDYARKIDELGGPQA---YFEKNLQARHEQLANSDGLRKMLADLQAGWNASSVIG
ApxIA   414FERVATKLANKIDEWEKKHG-KNYFENGYDARHSAFLEDT--FELLSQYNKEYSVERVVA
LtxA    419FEHIANQLADKIKAWENKYG-KNYFENGYDARHSAFLEDS--LKLFNELREKYKTENILS
AqxA    425FEHVATKLSGKIEAWEKKYG-MNYFEKGYDARHAAFLEDN--FAFFESLTKELRAERVIS
RtxA    413FESVANRLQSKILAWEKENGGKNYFENGYDARHAHYLERN--LKLLSELNKELQAERVIA
HlyA    418FEHVASKMADVIAEWEKKHG-KNYFENGYDARHAAFLEDN--FEILSQYNKEYSVERSVL

           VQTTEISKSALELAAITGNADNLKSVDVFVDRFVQGE-----RVAGQPVVLDVAAGGIDI
           ITQQRWDVNIGELAGITRKGSDTKSGKAYVDFFEEGKLLEKEPDRFDKKVFDPLEGKIDL
           ITQQGWDQRIGELAGITRNGDRIQSGKAYVDYLKKGEELAKHSDKFTKQILDPIKGNIDL
           ITQQQWDTQIGDLAGITRRGDKIQSGKAYVDVFKETK---KDLSYDNIVTFDPTEGIIDI
           ITQQRWDANIGELAGITKLGDRISSGKAYADAFEDGK----KLDGASNVTVDTRTGVVDI
           ITQQHWDTLIGELAGVTRNGDKTLSGKSYIDYYEEGKRLEKKPDEFQKQVFDPLKGNIDL

           ASRKG-ERPALTFITPLAAPGEEQRRRTKTGKSEFTTFVEIVGKQDRWRIRDG-AADTTI
           SSIN--KTTLLKFVTPVFTAGEEIRERKQTGKYEYMTELFVKGK-EKWVVTGVQSHNAIY
           SGIK--GSTTLTFLNPLLTAGKEERKTRQSGKYEFITELKVKGR-TDWKVKGVPNSNGVY
           SKTTS-KTQHLLFLNPLLTPGKENREREKKGKYEYVTKLIVDRK-TKWQVTDG-EASSTL
           SNANGKKTQALHFTSPLLTAGTETRERVQNGKYSYINQLKFNRV-KSWTVKDG-EANSRL
           SDSK--SSTLLKFVTPLLTPGEEIRERRQSGKYEYITELLVKGV-DKWTVKGVQDKGAVY

           DLAKVVSQLVDAN-----GVLKHSIKLDVIGGDGDDVVLANASRIHYDGGAGTNTVSYAA
           DYTNLIQLAID------KKGEKRQVTIESHLGEKNDRIYLSSGSSIVYAGNGHDVAYYDK
           DFSNLIQHAVT------RDNKVLEARLIANLGAKDDYVFVGSGSTIVNAGDGYDVVDYSK
           DFTNVLQFIAVDTDRAGNVTESLEAKIEAKLGKGDDTVFVGWGSTDIDGGEGYDRAAYNR
           DFSKVIQHVAFNDEDGRLSGKTEEIALNVNAGSGNDDIFAGQGKMNVDGGTGHDRVFYSK
           DYSNLIQHASV------GNNQYREIRIESHLGDGDDKVFLSAGSANIYAGKGHDVVYYDK

           LG--------RQDSITVSADGERFNVRKQLNN-ANVYREGVATQTTAYGKR-TENVQYRH992
           TD----TGYLTFDGQSAQKAGE-YIVTKELKADVKVLKEVVKTQDISVGKTCSEKLEYRD696
           GR----TGALTIDGRNATKAGQ-YKVERDLSG-TQVLQETVSKQETKRGKV-TDLLEYRN696
           LDGEAHIYGLNIDAQQETVAGS-YTVNRTIAG-GAAKHEVIKVHQATAGKR-VDKIEYRQ712
           DGG---LGQVNVDGTKATEAGS-YTVNRSINN-GSFYHEVIKRQTTQVGKR-TETLEYRD698
           TD----TGYLTIDGTKATEAGN-YTVTRVLGGDVKVLQEVVKEQEVSVGKR-TEKTQYRS699
```

Fig. S1. ClustalW sequence alignment of a partial sequence of the acylated segments of RTX toxins. CyaA, *Bordetella pertussis* (UniProtKB P0DKX7); ApxIA, *Actinobacillus pleuropneumonia* (UniProtKB P55128); LtxA, *Aggregatibacter actinomycetemcomitans* (UniProtKB P16462); AqxA, *Actinobacillus equuli* (UniProtKB Q8KWZ9); RtxA, *Kingella kingae* (UniProtKB A0A1X7QMH9); HlyA, *Escherichia coli* (UniProtKB A0A4Z0T8K2). The conserved aromatic residues are highlighted in red, and lysines modified by fatty acyl are highlighted in green.

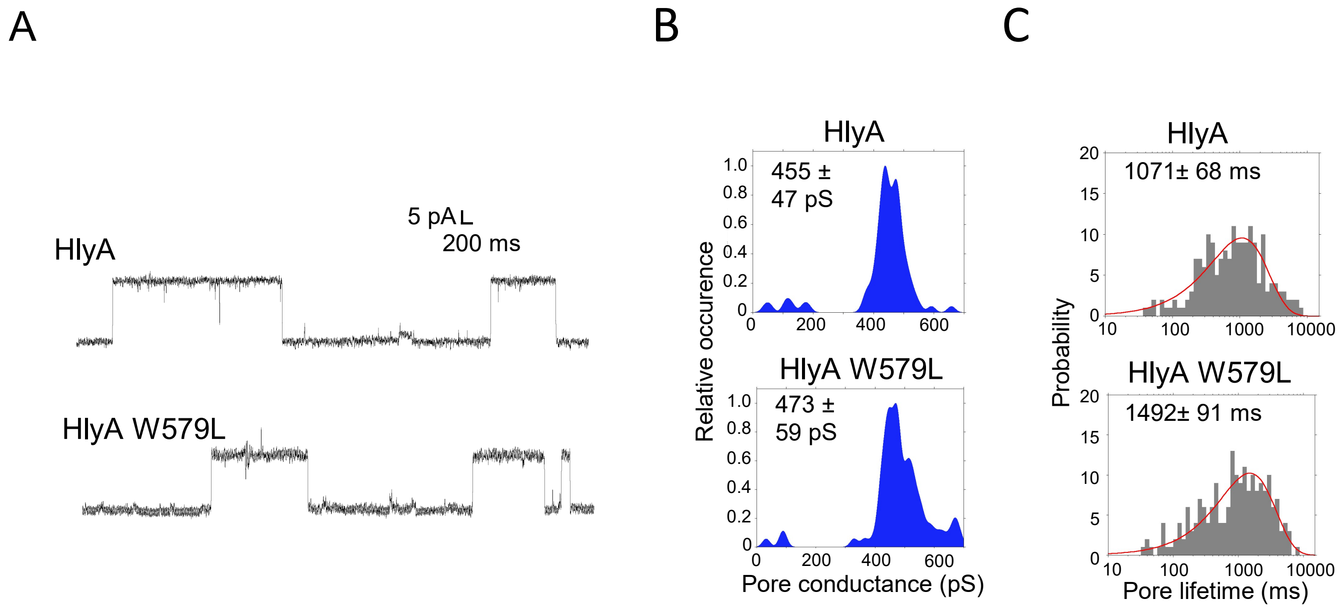

Fig. S2. The pore conductance and pore lifetime of individual pores formed by HlyA variants were similar. (A) Single-pore recordings of asolectin membranes in the presence of 500 pM purified HlyA. Conditions: 50 mM KCl, 10 mM Tris-HCl (pH 7.4), 2 mM $CaCl_2$; applied voltage was 50 mV; temperature was 25 °C, and recording was filtered at 10 Hz. **(B)** Kernel density estimation (KDE) of single-pore conductances calculated from single-pore recordings (> 500 events) acquired on different asolectin membranes with 500 pM HlyA variants under the same conditions as in A. **(C)** To determine lifetimes, > 250 single pore openings were recorded on different asolectin membranes with 500 pM concentrations of HlyA variants under the same conditions as in A.

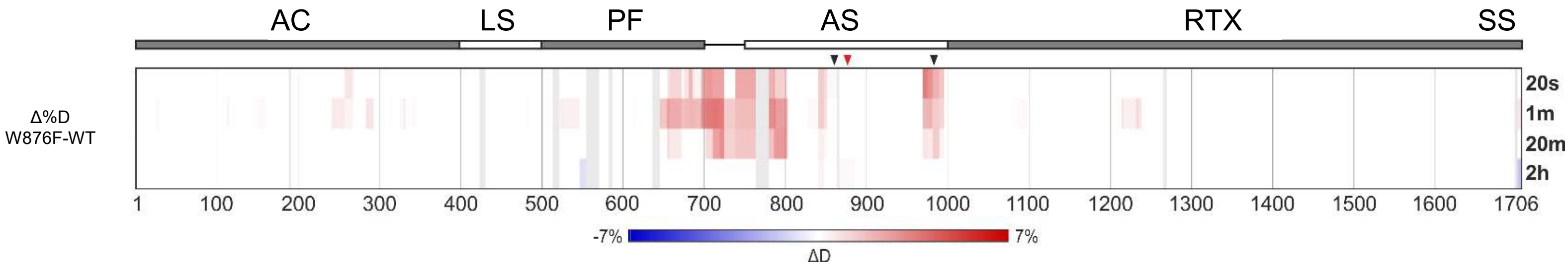

Fig.S3. Differential heat map visualizing difference in deuteration level between the wild-type CyaA and CyaA W876F. Deuteration level of the wild-type protein was subtracted from that of the W876F mutant. Changes in deuteration are visualized by the blue (protection/lower deuteration)-white (no change)-red (deprotection/higher deuteration) gradient. Domains are depicted above the heat map as well as the positions of acylation (black arrowheads) and the W876F site (red arrowhead). Regions in grey were not covered by the HDX data. AC, AC domain; LS, linker segment; PF, pore-forming domain, AS, acylated segment; RTX, RTX domain ; SS, secretion signal.

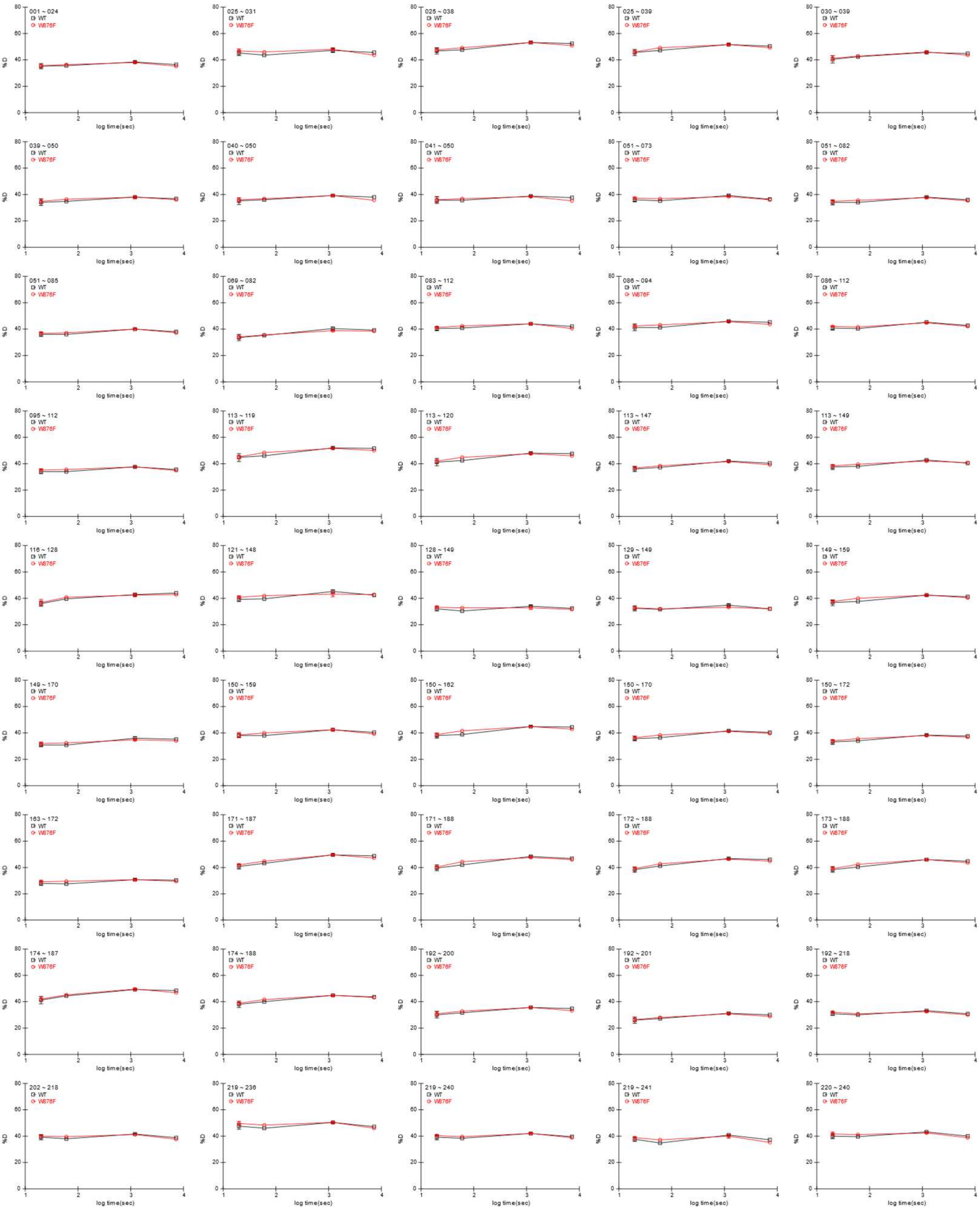
001 ~ 024
025 ~ 031
025 ~ 038
025 ~ 039
030 ~ 039
039 ~ 050
040 ~ 050
041 ~ 050
051 ~ 073
051 ~ 082
051 ~ 085
069 ~ 082
083 ~ 112
086 ~ 094
086 ~ 112
095 ~ 112
113 ~ 119
113 ~ 120
113 ~ 147
113 ~ 149
116 ~ 128
121 ~ 148
128 ~ 149
129 ~ 149
149 ~ 159
149 ~ 170
150 ~ 159
150 ~ 162
150 ~ 170
150 ~ 172
163 ~ 172
171 ~ 187
171 ~ 188
172 ~ 188
173 ~ 188
174 ~ 187
174 ~ 188
192 ~ 200
192 ~ 201
192 ~ 218
202 ~ 218
219 ~ 236
219 ~ 240
219 ~ 241
220 ~ 240
WT
W876F
%D
log time(sec)

Fig. S4-A.

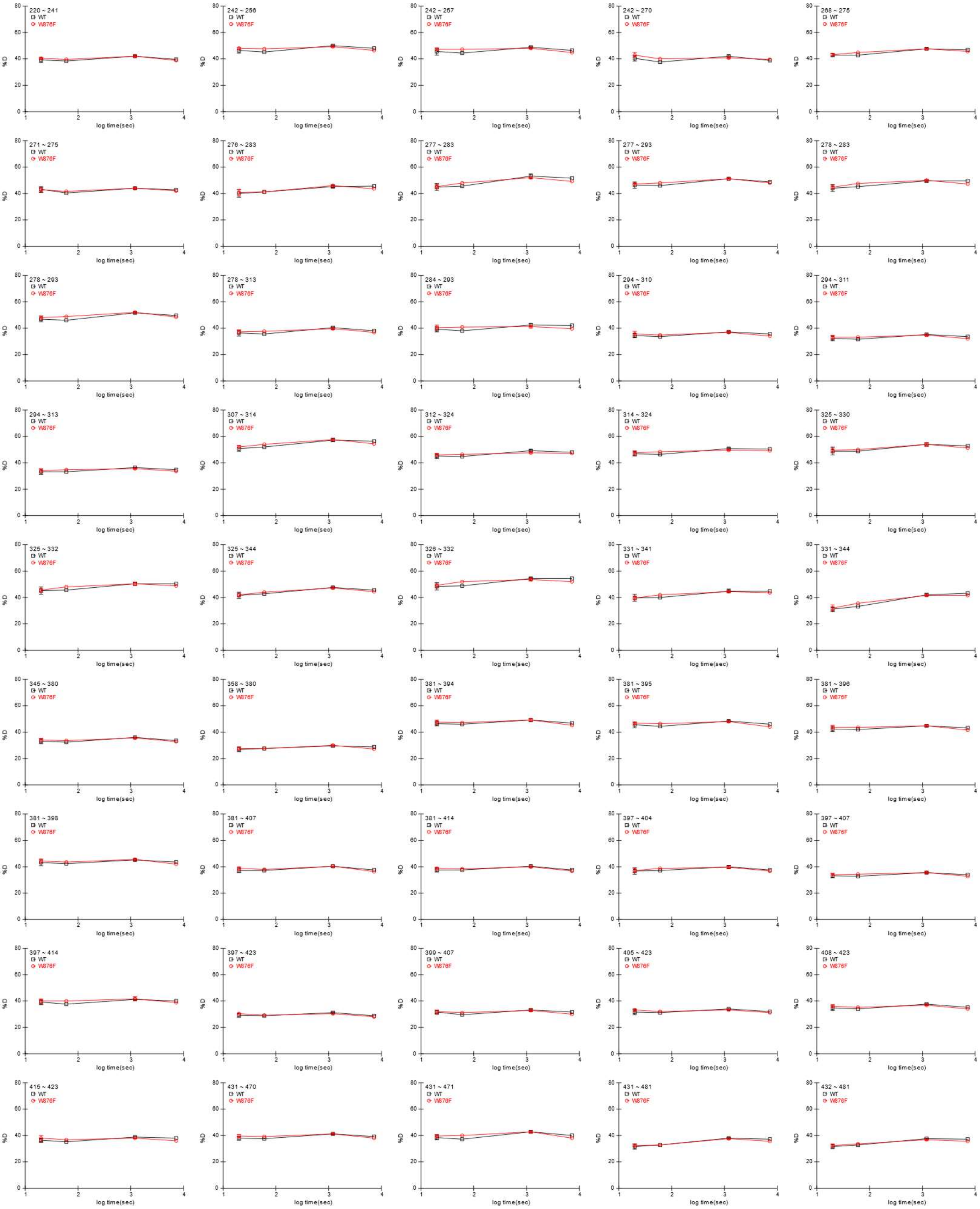
220 ~ 241
242 ~ 256
242 ~ 257
242 ~ 270
268 ~ 275
271 ~ 275
276 ~ 283
277 ~ 283
277 ~ 293
278 ~ 283
278 ~ 293
278 ~ 313
284 ~ 293
294 ~ 310
294 ~ 311
294 ~ 313
307 ~ 314
312 ~ 324
314 ~ 324
325 ~ 330
325 ~ 332
325 ~ 344
326 ~ 332
331 ~ 341
331 ~ 344
345 ~ 380
358 ~ 380
381 ~ 394
381 ~ 395
381 ~ 396
381 ~ 398
381 ~ 407
381 ~ 414
397 ~ 404
397 ~ 407
397 ~ 414
397 ~ 423
399 ~ 407
405 ~ 423
408 ~ 423
415 ~ 423
431 ~ 470
431 ~ 471
431 ~ 481
432 ~ 481
WT
W876F
%D
log time(sec)

Fig. S4-B.

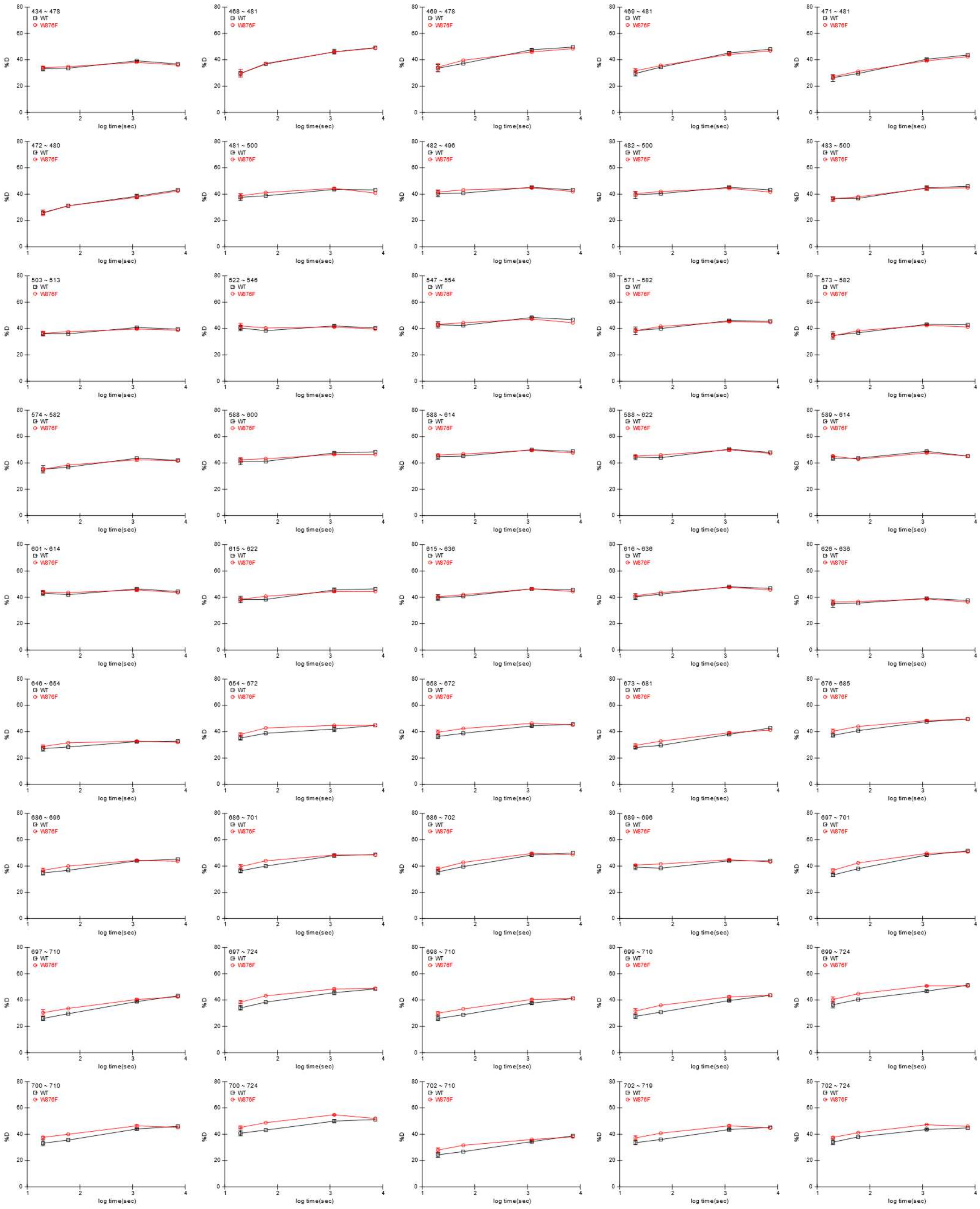
434 ~ 478
468 ~ 481
469 ~ 478
469 ~ 481
471 ~ 481
472 ~ 480
481 ~ 500
482 ~ 496
482 ~ 500
483 ~ 500
503 ~ 513
522 ~ 546
547 ~ 554
571 ~ 582
573 ~ 582
574 ~ 582
588 ~ 600
588 ~ 614
588 ~ 622
589 ~ 614
601 ~ 614
615 ~ 622
615 ~ 636
616 ~ 636
626 ~ 636
646 ~ 654
654 ~ 672
658 ~ 672
673 ~ 681
676 ~ 685
686 ~ 696
686 ~ 701
686 ~ 702
689 ~ 696
697 ~ 701
697 ~ 710
697 ~ 724
698 ~ 710
699 ~ 710
699 ~ 724
700 ~ 710
700 ~ 724
702 ~ 710
702 ~ 719
702 ~ 724
WT
W876F
%D
log time(sec)

Fig. S4-C.

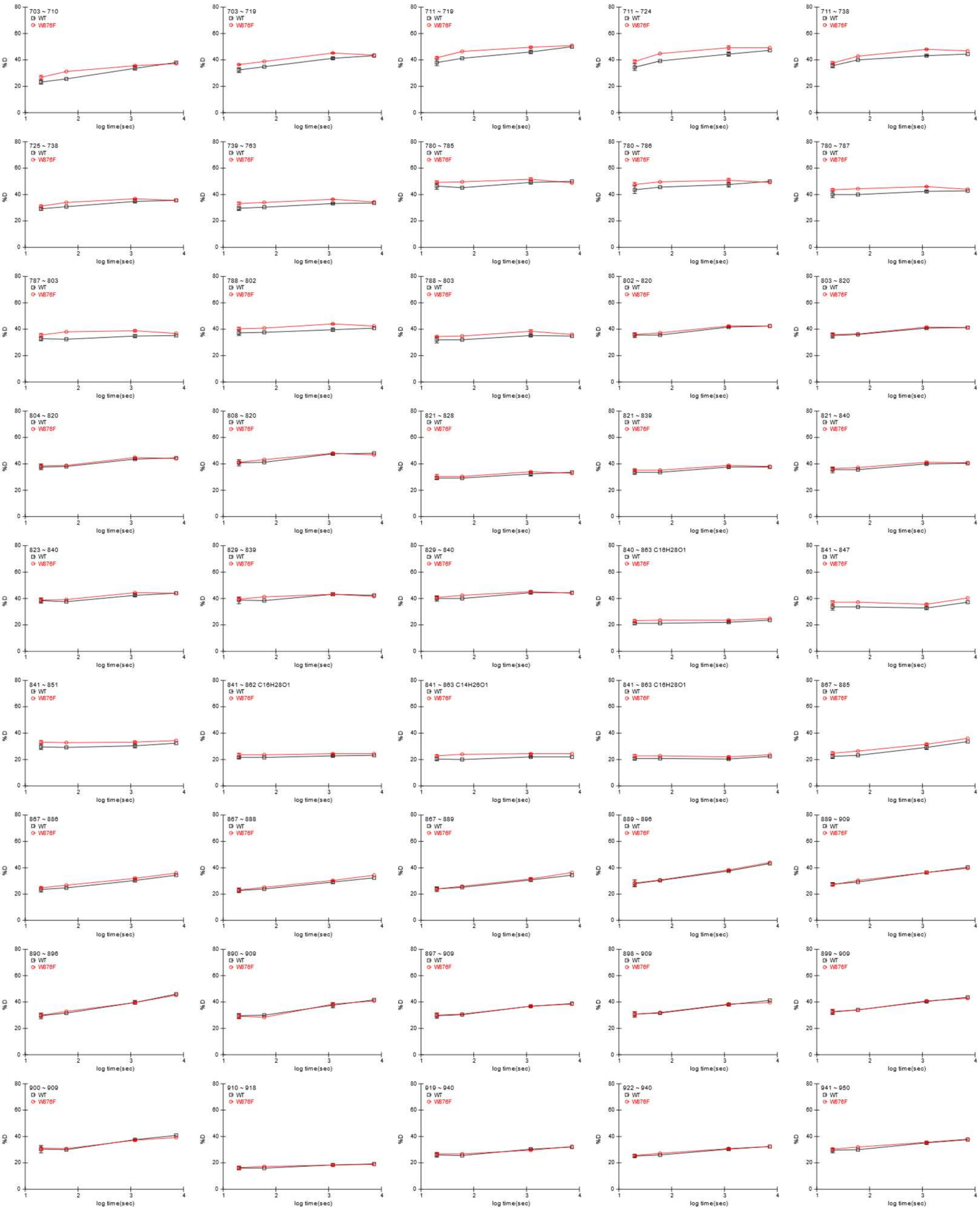
703 ~ 710
703 ~ 719
711 ~ 719
711 ~ 724
711 ~ 738
725 ~ 738
739 ~ 763
780 ~ 785
780 ~ 786
780 ~ 787
787 ~ 803
788 ~ 802
788 ~ 803
802 ~ 820
803 ~ 820
804 ~ 820
808 ~ 820
821 ~ 828
821 ~ 839
821 ~ 840
823 ~ 840
829 ~ 839
829 ~ 840
840 ~ 863 C16H28O1
841 ~ 847
841 ~ 851
841 ~ 862 C16H28O1
841 ~ 863 C14H26O1
841 ~ 863 C16H28O1
867 ~ 885
867 ~ 886
867 ~ 888
867 ~ 889
889 ~ 896
889 ~ 909
890 ~ 896
890 ~ 909
897 ~ 909
898 ~ 909
899 ~ 909
900 ~ 909
910 ~ 918
919 ~ 940
922 ~ 940
941 ~ 950
WT
W876F
%D
log time(sec)

Fig. S4-D.

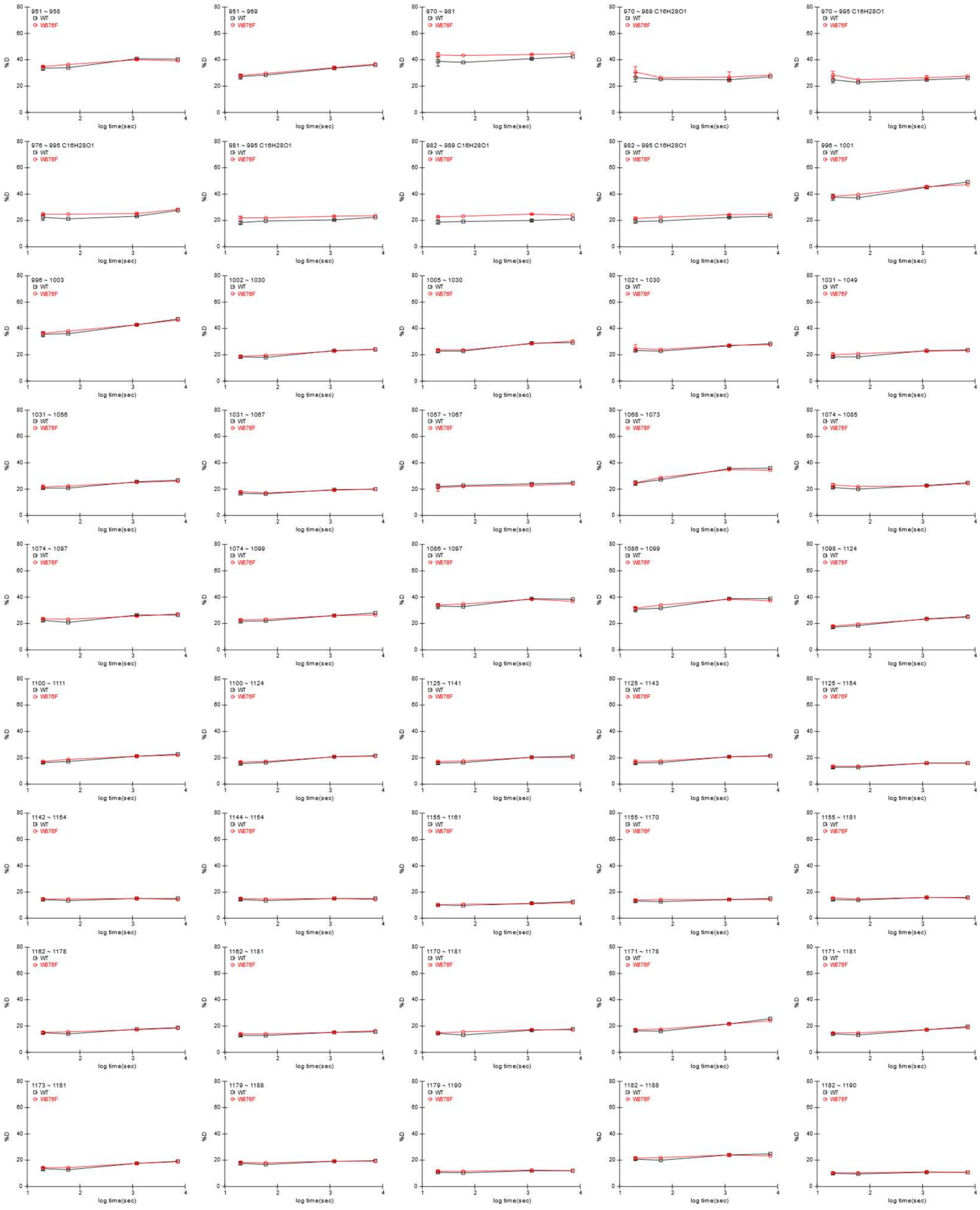
951 ~ 958
951 ~ 969
970 ~ 981
970 ~ 989 C16H28O1
970 ~ 995 C16H28O1
976 ~ 995 C16H28O1
981 ~ 995 C16H28O1
982 ~ 989 C16H28O1
982 ~ 995 C16H28O1
996 ~ 1001
996 ~ 1003
1002 ~ 1030
1005 ~ 1030
1021 ~ 1030
1031 ~ 1049
1031 ~ 1056
1031 ~ 1067
1057 ~ 1067
1068 ~ 1073
1074 ~ 1085
1074 ~ 1097
1074 ~ 1099
1086 ~ 1097
1086 ~ 1099
1098 ~ 1124
1100 ~ 1111
1100 ~ 1124
1125 ~ 1141
1125 ~ 1143
1125 ~ 1154
1142 ~ 1154
1144 ~ 1154
1155 ~ 1161
1155 ~ 1170
1155 ~ 1181
1162 ~ 1178
1162 ~ 1181
1170 ~ 1181
1171 ~ 1178
1171 ~ 1181
1173 ~ 1181
1179 ~ 1188
1179 ~ 1190
1182 ~ 1188
1182 ~ 1190
WT
W876F
%D
log time(sec)

Fig. S4-E.

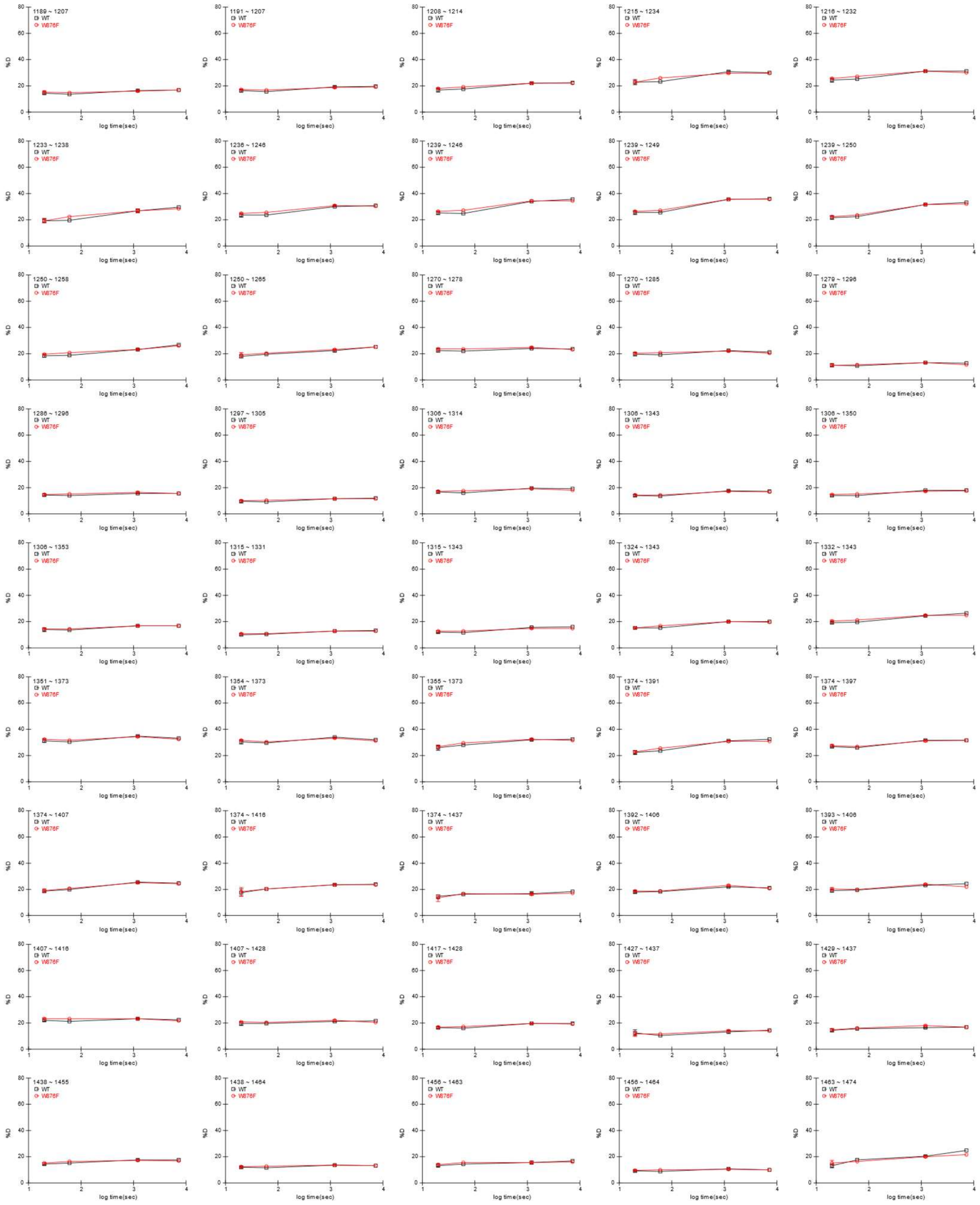
1189 ~ 1207
1191 ~ 1207
1208 ~ 1214
1215 ~ 1234
1216 ~ 1232
1233 ~ 1238
1236 ~ 1246
1239 ~ 1246
1239 ~ 1249
1239 ~ 1250
1250 ~ 1258
1250 ~ 1265
1270 ~ 1278
1270 ~ 1285
1279 ~ 1296
1286 ~ 1296
1297 ~ 1305
1306 ~ 1314
1306 ~ 1343
1306 ~ 1350
1306 ~ 1353
1315 ~ 1331
1315 ~ 1343
1324 ~ 1343
1332 ~ 1343
1351 ~ 1373
1354 ~ 1373
1355 ~ 1373
1374 ~ 1391
1374 ~ 1397
1374 ~ 1407
1374 ~ 1416
1374 ~ 1437
1392 ~ 1406
1393 ~ 1406
1407 ~ 1416
1407 ~ 1428
1417 ~ 1428
1427 ~ 1437
1429 ~ 1437
1438 ~ 1455
1438 ~ 1464
1456 ~ 1463
1456 ~ 1464
1463 ~ 1474
WT
W876F
%D
log time(sec)

Fig. S4-F.

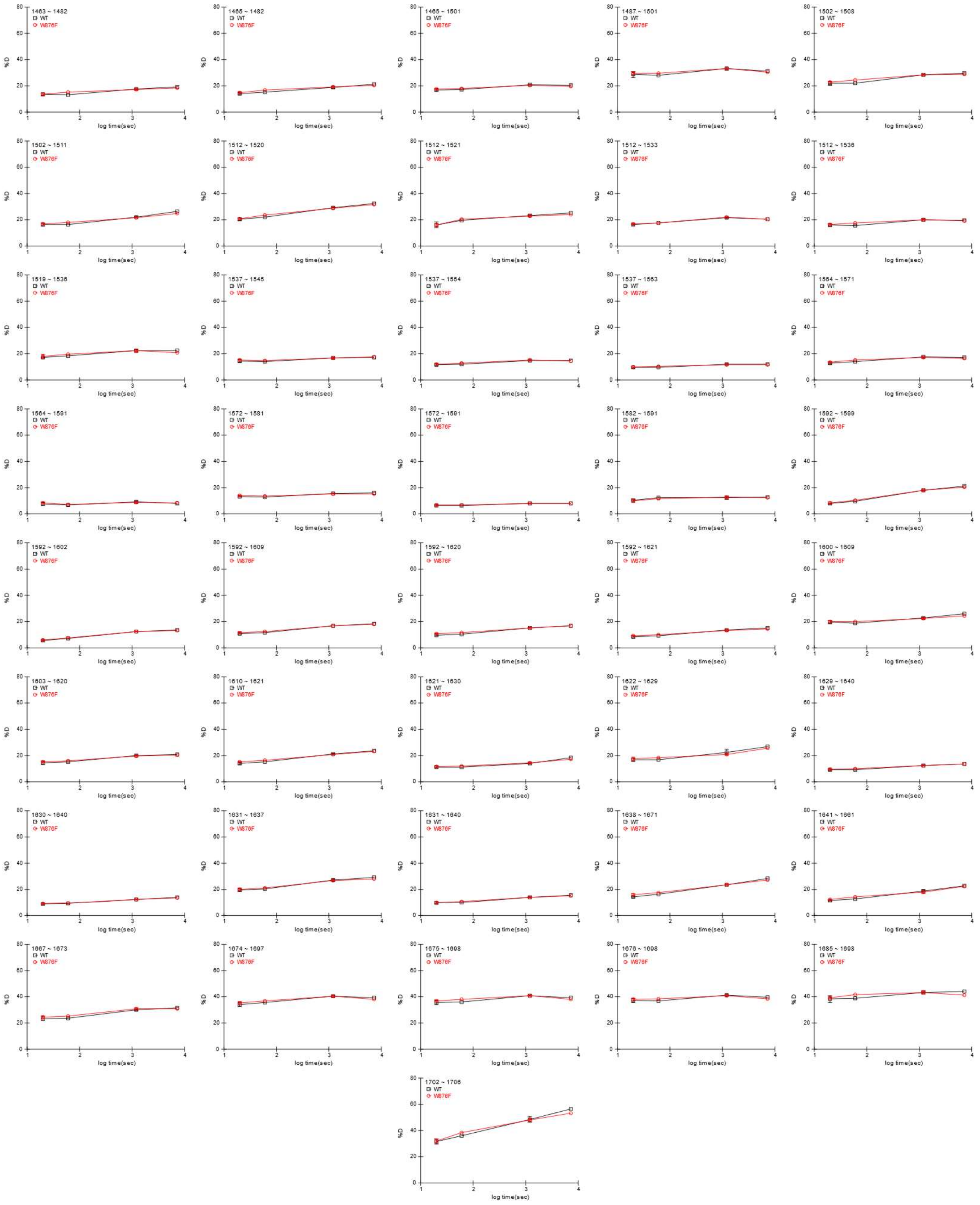

Fig. S4 G.

Figs S4 A-G. Deuterium uptake plots for all peptides covering entire sequence of CyaA. Black – wild type, red – W876F mutant. Time points 20sec and 20min were triplicated and average values with respective s.d. are shown. Peptide limits in native sequence numbering are indicated in the top left corner of each graph.

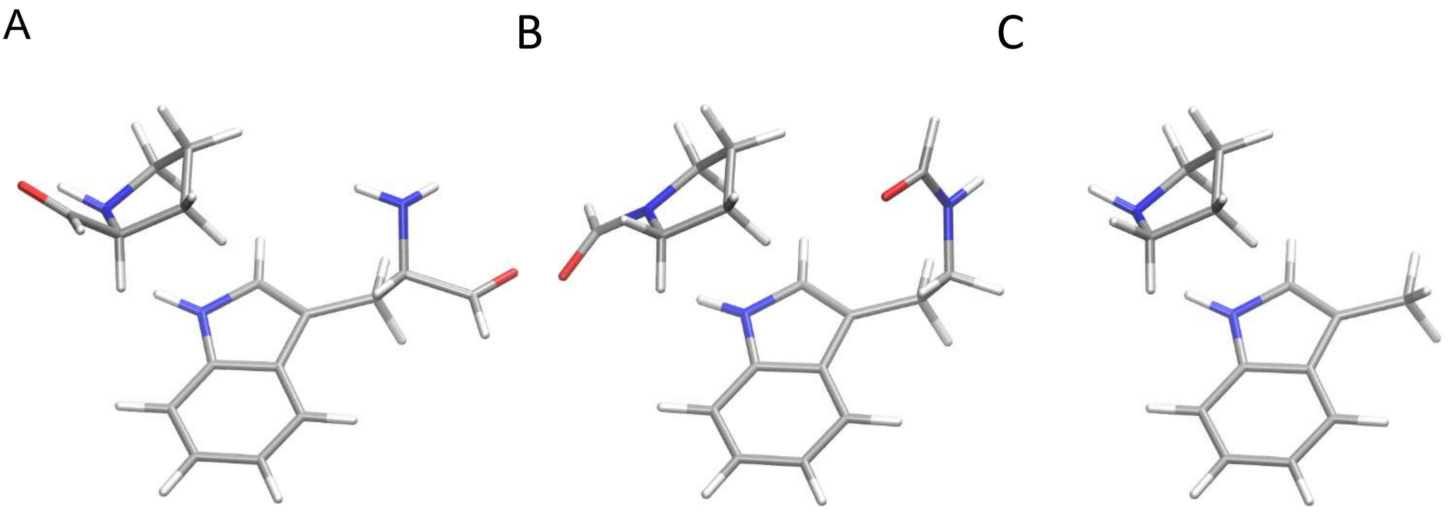

Fig. S5. Three different model complexes used to represent the interaction of residues Trp876 and Pro848 in CyaA, created in Visual Molecular Dynamics software. (A) “trp-pro” model, (B) “large ” model (C) “small ” model. The grey, red, blue and white colors indicate carbon, oxygen, nitrogen and hydrogen atoms, respectively.

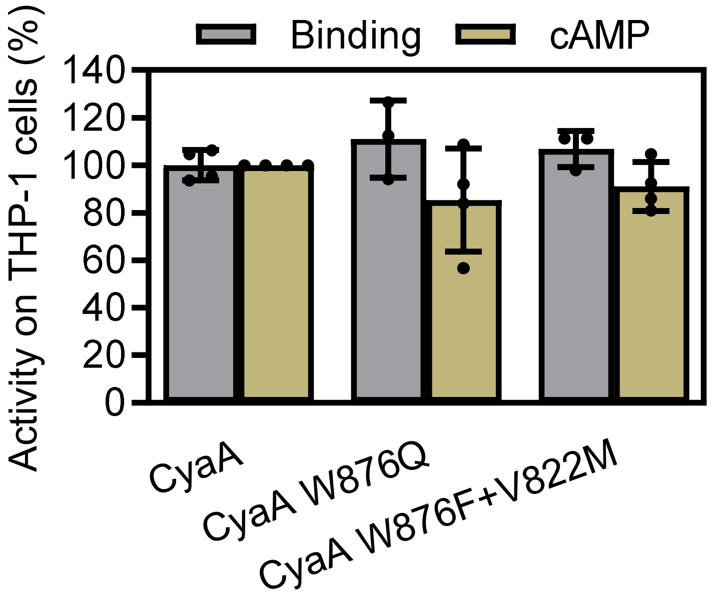

Fig. S6. The capacity of the CyaA variants to bind and intoxicate CR3-expressing THP-1 monocytes remained intact. Cell binding and cAMP intoxication of CyaA variants was determined on human CR3-expressing THP-1 monocytes. Toxin binding to THP-1 was determined as the amount of total cell-associated AC enzyme activity after incubation of 10^6 cells with 1 μg/ml of the protein for 30 min at 4°C. AC translocation was assessed by determining the intracellular cAMP concentration by ELISA as described in Experimental Procedures. Activities are expressed as a percentage of CyaA activity and represent the means ± SD of three independent determinations.

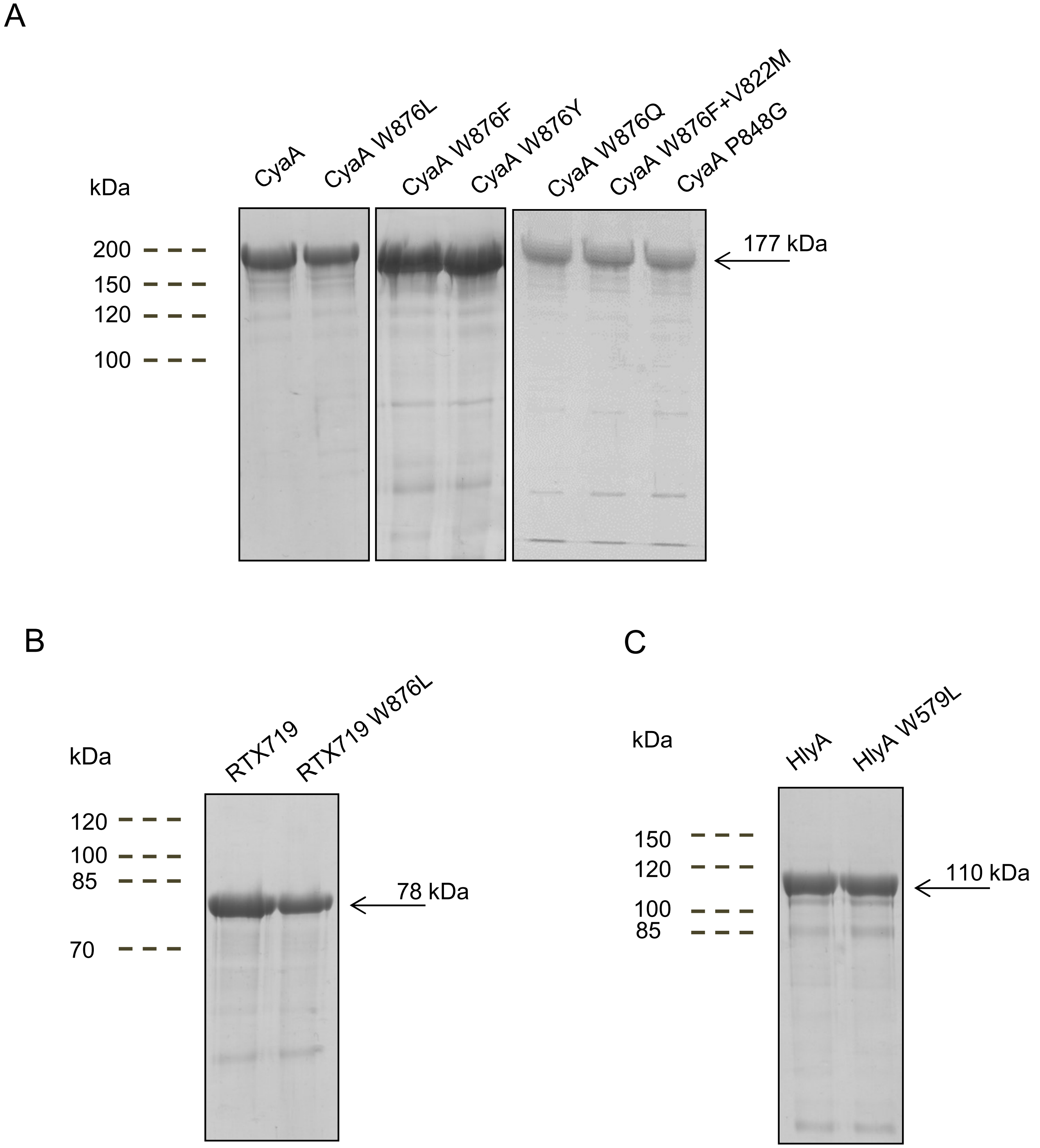

Fig. S7. SDS PAGE analysis of purified CyaA and HlyA variants. CyaA **(A),** CyaA-derived RTX719 **(B)**, and HlyA **(C)** variants were expressed in *E. coli* XL-1 Blue, and proteins were purified close to homogeneity from urea-solubilized inclusion bodies by two-step chromatography on DEAE and Phenyl-sepharose (CyaA variants), DEAE-Sepharose (RTX719 variants), or NiNTA agarose (HlyA variants). Samples were analyzed on 7.5% polyacrylamide gels and stained with Coomassie Blue.
